# Tropical cyclone impact data in the Philippines: implications for disaster risk research

**DOI:** 10.1007/s11069-025-07394-x

**Published:** 2025-06-20

**Authors:** Elizabeth G. Galloway, Jennifer L. Catto, Chunbo Luo, Stefan Siegert

**Affiliations:** 1 UKRI Centre for Doctoral Training in Environmental Intelligence: Data Science and AI for Sustainable Futures, University of Exeter, Exeter, UK; 2 Department of Mathematics and Statistics, University of Exeter, Exeter, UK; 3 Department of Computer Science, University of Exeter, Exeter, UK

**Keywords:** Tropical cyclones, Impact data, Utility, Risk, Applications

## Abstract

Natural hazards such as tropical cyclones (TCs) cause widespread destruction. Historical impact data provides a resource for understanding TC impacts and associated societal vulnerabilities which is essential for building resilience. However, characteristics of impact data such as resolution and coverage can influence its utility for disaster risk reduction (DRR) applications. With this in mind, we present a province-level impact dataset for TCs in the Philippines between 2010 and 2020 for deaths, affected population, housing damage and economic loss curated for DRR applications. Specifically, we evaluate the effect of the dataset’s spatial resolution and its coverage of hazard intensities, impact magnitudes and impact types and discuss the implications for DRR applications. Considering the utility of impact data within the context of DRR is crucial, and a dataset with comprehensive coverage of impact and hazard magnitudes and appropriate spatial resolution is pivotal for DRR applications. The research presents a guide for others using this dataset and data more generally in DRR applications.

**Supplementary Information:**

The online version contains supplementary material available at 10.1007/s11069-025-07394-x.

## Introduction

### Background

Impact data provides us with a record of the effects of historical natural hazards, for example the number of people displaced, houses damaged, or the cost of repairs during or following an event. This record is pivotal to many disaster risk reduction (DRR) applications as analysing historical impacts can provide fundamental insights into disaster risk and allows DRR researchers to assess the risk of impacts from current or future hazards (United Nations Office for Disaster Risk Reduction (UNDRR) 2015; Wirtz et al. 2014). The IPCC defines “risk” as “the potential for adverse consequences” to “human or ecological systems” (Reisinger et al. 2020). Disaster risk arises from interactions between hazard, exposure, and vulnerability characteristics (Blaikie et al. 2014; Lavell et al. 2012), which are often analysed during disaster risk assessments (e.g. (Quesada-Román 2022; Cadiz 2018)). Spatial, temporal and statistical analyses of historical impact data can also be used to assess trends in disaster impacts across regions and through time (Aksha et al. 2018; Ali et al. 2022; Kaiser et al. 2021; Marulanda et al. 2010; Brennan and Danielak 2022; Lou et al. 2012; Choi et al. 2019; Lin et al. 2021; Luu and von Meding 2019; Aksha et al. 2020; Santos 2021; Cinco et al. 2016; Quesada-Román and Campos-Durán 2023; Quesada-Román et al. 2024). These applications offer valuable support to decision makers considering disaster management strategies, but for these to be effective, the context of the impact data and the complexity of risk landscapes must be considered (United Nations Office for Disaster Risk Reduction (UNDRR) 2022). Although open impact data sources are important for accessible and reproducible impact-based research, they are known to suffer from several shortcomings and data biases (Gall et al. 2009; Jones et al. 2022; Koç and Thieken 2018; Wyatt et al. 2023; Moriyama et al. 2018; Osuteye et al. 2017; United Nations Office for Disaster Risk Reduction (UNDRR) 2022; Mazhin et al. 2021). Specifically, given that impact data from different sources differ in accuracy, coverage, and resolution, it is important to consider the key factors influencing the utility of the data within the research-specific context (Romão and Paupério 2016; Wyatt et al. 2023). This allows researchers to use impact data in alignment with the scope of the research question whilst minimising the effects of bias (Miller et al. 2022). This could involve identifying new data opportunities, improving, or combining existing data sources such that the value of the data is maximised within DRR research and its applications. In this paper we present a dataset of the impacts of tropical cyclones (TCs) in the Philippines that has been curated with consideration for DRR research requirements, presenting insights from the dataset that demonstrate the utility and benefits of high-resolution, high-coverage impact data.

### The limitations of impact data for DRR research

Insufficient resolution, incomplete spatial and temporal coverage, and the omission of low-impact events can all affect the utility of impact data for DRR research applications. It is common for impact data from open sources to be low in resolution, often as low as country-level (Mazhin et al. 2021; Moriyama et al. 2018). At low resolutions, the finer-scale details which represent crucial aspects of disaster risk can be dampened and can fail to capture the spatial heterogeneities of most natural hazard events, let alone the physical and socioeconomic processes underlying the impacts themselves (Enenkel et al. 2020; Moriyama et al. 2018). However, several studies which have been able to make use of sub-national impact data have demonstrated its ability to capture heterogeneity in exposure and vulnerability to natural hazards and improve our understanding of vulnerability compared to global impact data sources (Rosvold and Buhaug 2021; Aksha et al. 2018; Lin et al. 2021).

The coverage, or completeness, of an impact dataset affects its ability to represent disaster risk comprehensively. Whilst the historical impact data record is undeniably valuable, even the longest records are often short in comparison to the return periods of extreme natural hazards (Woo 2019). For example, the return period of Typhoon Haiyan, which caused extensive destruction in the Philippines in 2013, was estimated to be 200 years (Takagi and Esteban 2016). However, many DRR applications rely on a complete record of disaster impacts to create useful outputs. Applications such as impact-based forecasting or risk mapping which are underpinned by historical impact data can suffer from inaccuracies if the data contains gaps in space, time, or in the magnitude of hazards and their corresponding impacts (Merz et al. 2020). In particular where historical impact data influences decisions surrounding early warnings and mitigation, it is critical for the dataset to accurately reflect real-world risk to disaster events.

Thresholds are often imposed in disaster loss databases below which events are not included in the database, which can lead to incomplete or unbalanced datasets with respect to low-impact and small-scale events (Gall et al. 2009; Moriyama et al. 2018; Brennan and Danielak 2022; United Nations Office for Disaster Risk Reduction (UNDRR) 2019). Whilst most DRR research focuses on large-scale, high-impact events, literature focusing specifically on small-scale or low-impact events has demonstrated their importance, particularly for socioeconomically vulnerable communities (Marulanda et al. 2010). For example, in a study of impact data for Senegal and Mali, Brennan and Danielak find that small disasters account for the vast majority of events and have higher cumulative impacts for some impact types compared to large disasters (Brennan and Danielak 2022). In terms of DRR research, low-impact events could result from several scenarios which inform our understanding of risk and vulnerability. For example, a small community could suffer damage to a significant proportion of its houses due to highly vulnerable buildings, despite the total damage being below the reporting threshold. Alternatively, an event which would have been devastating in the past may result in a far smaller impact due to effective preparation measures. Therefore, in many DRR applications, low-impact events represent a valuable component of an impact dataset.

### Tropical cyclone impact data requirements

TCs are among the most destructive natural hazards on the planet, causing nearly 780,000 deaths and economic losses of $1.4 trillion between 1970 and 2019 (World Meteorological Organization (WMO) 2021). TCs are multi-hazard events, often triggering flooding, storm surges, landslides and wind damage, leading to impacts across different spatial scales. To use historical impact data to represent TC risk comprehensively, the data must be able to capture both the overall footprint of the storm system (which can be hundreds of miles in radius), and the effects of their multiple hazards (which can occur on smaller, local scales). Additionally, since vulnerability to different sub-hazards is dependent on factors such as proximity to water bodies, elevation, land use, and socioeconomic features, disaggregating historical TC impact data between regions can be highly useful in highlighting vulnerability differences (World Meteorological Organization (WMO) 2021; United Nations Office for Disaster Risk Reduction (UNDRR) 2015). The variety of spatial scales involved in TC impacts means that the completeness of impact records is particularly important; thresholds which exclude low-impact or small-scale TC events means that the impacts of TC events with localised sub-hazards such as landslides or flash floods may not be captured. Further, the nature of TC evolution means that some areas are prone to frequent, low-intensity TCs with low impacts that could similarly be excluded by thresholds.

Since TC activity can be highly variable between years and is subject to influence by global-scale teleconnections, both TC frequency and intensity can change depending on the selected time period (Cinco et al. 2016; Lin et al. 2020; Corporal-Lodangco et al. 2016). As such, TC-related impacts measured in different years can be highly variable, making it important to consider the time period of a historical TC-related impact dataset and whether its coverage is appropriate for the research task.

### Setting

The Philippines is ranked as the country with the highest disaster risk worldwide according to the World Risk Index (Institute for International Law of Peace and Armed Conflict (IFHV) 2023, 2022). The Philippines’ meteorological setting within the Northwest Pacific Ocean and adjacent to the Philippine Sea places the country within a highly active Tropical Cyclone (TC) belt (Center for Excellence in Disaster Management and Humanitarian Assistance (CFE-DM) 2021; Yumul et al. 2011). In an analysis of over 60 years of TC data preceding 2013, Cinco et al. found that an average of 19.4 TCs entered the Philippine Area of Responsibility (PAR) each year, nine of which made landfall on average (Cinco et al. 2016). TCs frequently displace populations and damage the built and natural environments in the Philippines, in the worst cases threatening the lives and livelihoods of citizens (Lagmay and Racoma 2019; Santos 2021). Climate model projections suggest that the proportion and intensity of the strongest TCs will increase under climate change (Knutson et al. 2020; Gallo et al. 2019; Cha et al. 2020). Further, factors such as increased intensity and sea-level rise are expected to drive more intense TC precipitation, increasing the risk of TC-induced flooding (Marsooli et al. 2019; Xi and Lin 2022). It is therefore becoming increasingly important to understand TC impacts and build resilience in the Philippines (Intergovernmental Panel on Climate Change (IPCC) 2019; Gallo et al. 2019).

In addition to the complexity of TC impacts, the Philippines also exhibits significant heterogeneity in geographical and socioeconomic conditions between its regions and provinces (Baldwin et al. 2023). This superposition of risk from different sources on different spatial scales suggests that sub-national, detailed impact data could reveal important patterns in past and present vulnerability to TCs. Such knowledge can help inform future protective measures against TC impacts and by building resilience, extensive societal benefits such as food security, education and equality can be promoted (United Nations Office for Disaster Risk Reduction (UNDRR) 2015).

### Data source and previous work

The Philippines’ National Disaster Risk Reduction and Management Council (NDRRMC) publishes disaster loss data in a series of Situational Reports, providing a high-resolution, detailed record of impact data for stakeholders (National Disaster Risk Reduction and Management Operations Center 2021). The NDRRMC is made up of several member agencies, headed by the Department of National Defence, with functions and responsibilities implemented at national and sub-national levels aimed to support all aspects of the country’s disaster risk reduction and management (National Disaster Risk Reduction and Management Operations Center 2021). A key responsibility of the NDRRMC involves submitting situational reports for hazardous events that occur in the Philippines (National Disaster Risk Reduction and Management Operations Center 2021).

The high-resolution impact data covering a variety of impact types provided in the NDRRMC reports could address some of the shortfalls of open-source disaster loss databases and provide valuable insights into disaster risk in the Philippines. A handful of studies to date have used data from the NDRRMC reports to investigate the impacts of TCs in the Philippines, across a variety of time periods and temporal and spatial resolutions (Cinco et al. 2016; Lagmay and Racoma 2019; Santos 2021; Esteban et al. 2013; Yonson et al. 2018; Healey et al. 2023; Gray et al. 2022). Most studies utilising data from the NDRRMC reports either focus on one type of impact at a sub-national resolution or look across multiple impact types for a subset of TC events. The former approach is employed by Cinco et al., who use NDRRMC data to examine temporal trends in the economic cost of TC-related damages, aggregated over the whole country at an annual resolution (Cinco et al. 2016). The latter approach is taken by Lagmay and Racoma, who investigate two damaging storms in 2017, accounting the impacts of these at the province level for the areas of interest to the study (Lagmay and Racoma 2019). Similarly, Santos selects a subset of NDRRMC reports representing destructive storms in 2020, giving an account of multiple impact types for each storm and the regions affected, concluding by recommending a 10-year study of TCs (Santos 2021). These studies can highlight important location and event-specific risks, however, datasets that provide snapshots of TC impacts are unable to represent the complete TC risk landscape, limiting their value in some DRR applications. Esteban et al. make steps towards this, aiming to capture spatial trends in underlying physical and social conditions by splitting the country into five separate regions and analysing the relationships between TC wind speed and housing damage in each region for 22 individual TC events (Esteban et al. 2013). Yonson et al. use even higher resolution impact data, analysing the key determinants of province-level TC-related deaths between 2005 and 2010 (Yonson et al. 2018). Finally, Healey et al. identify trends at municipality level, investigating the relationship between housing vulnerability and TC-related deaths between 2005 and 2015, capitalising on the spatially disaggregated data provided in the reports (Healey et al. 2023). Similarly, Gray et al. analyse TC-related deaths at municipality level (Gray et al. 2022). These studies have demonstrated the ability of the NDRRMC data to identify patterns in impacts and begin to unravel the underlying relationships between the physical and socioeconomic settings of TC events.

Existing studies using NDRRMC impact data are limited to short time periods, single regions or impact types, or individual events. Here, we aim to expand the coverage of the existing body of research using NDRRMC impact data and demonstrate the benefits of doing so, aiming to facilitate and encourage new opportunities within DRR research. To do this, we present a high-resolution, spatially disaggregated TC impact dataset for the Philippines collected from the NDRRMC Situational Reports. The dataset includes fatalities, affected population, houses damaged and destroyed, and infrastructural and agricultural losses due to TCs between 2010 and 2020. This builds upon previous work which uses TC impact data in the Philippines by presenting and analysing a longer and more up-to-date time series of multiple impact types. First, the data collection process and resulting dataset features are outlined in Sect. 2. Evidence from the dataset is then presented to show its value within DRR applications and its ability to provide insight into TC vulnerability. Specifically, we assess the prevalence and importance of low-impact events in the dataset (Sect. 3.1.1). We also demonstrate that disaggregated, province-level impact data captures exposure to TC hazards and spatial patterns of vulnerability, and provides high coverage across impact and hazard magnitudes (Sect. 3.1.2 and 3.1.3). This further enables us to show different spatial and temporal patterns between the multiple impact types, reflecting the variable conditions across the country (Sect. 3.2).

## Methods

Here we detail the impact types selected and the process followed to collect the data and document the corresponding TC hazards.

### Selection of impact types

The selected impact types aim to reflect the multi-faceted nature of disaster losses by covering economic, human, and physical impacts, with particular attention paid to including impacts which shape lived experiences of disasters (Tschakert et al. 2017, 2019). This is balanced against the constraints of manual data collection which limits the number of variables it is possible to include. In addition, to create consistency and reduce bias between TC event impact records, it is only suitable to include impact types which are routinely published in the NDRRMC reports.

The final dataset includes the following impact types:

- Number of deaths;
- Number of people affected;
- Number of houses damaged;
- Number of houses destroyed;
- Infrastructural economic losses (in PhP: Philippine peso);
- Agricultural and fisheries economic losses (in PhP: Philippine peso) (referred to as ‘agricultural losses’ herein).

The National Disaster Risk Reduction and Management Operations Center “Standard Operating Procedures and Guidelines” outlines Situational Report requirements and reporting procedures, where information reported and verified at sub-national levels is consolidated into a single report, and provides definitions and descriptions of key impact types (National Disaster Risk Reduction and Management Operations Center 2021). In these guidelines, “Affected Population” is defined as “Individuals and families who were devastated by the impact of disasters, whether physical harm/damage befell upon them or their properties and whose daily functions are interrupted by the disaster”. Houses damaged, or “Partially Damaged Houses” as referred to in the guidelines, are defined as “Livable with reusable shelter materials and/or with the existing/remaining features based on its original structure”. Houses destroyed, or “Totally Damaged Houses”, refers to houses which are “Entirely destroyed and unfit for habitation or without any of the structural features indicated on the partially damaged”. The guidelines also outline some examples of infrastructure, such as roads and bridges, and agriculture, such as crops and equipment, which are considered during a damage assessment.

By including four separate indicators of physical damage to the built and natural environment (agricultural and infrastructural economic losses, houses damaged and houses destroyed), we also aim to capture the physical destructiveness of each TC. We acknowledge that whilst this is not a perfect representation of the wide-ranging types of impacts caused by TCs, it captures a more complete overview of disaster risk than a single impact type and takes into consideration the chain of effects that can be physically and socially damaging.

### Data collection

The data for this study are documented in the NDRRMC Situational Reports (‘SitReps’ herein) which give a brief meteorological history followed by a mix of text and tabulated data describing the impacts of TC events which have affected the Philippines (National Disaster Risk Reduction and Management Operations Center 2021). TC events which impacted the Philippines within the study period are identified through the NDRRMC archives and compared to events listed in other openly available sources, primarily the EM-DAT database (EM-DAT, CRED / UCLouvain, Brussels, Belgium - www.emdat.be), to avoid missing TC events in error. The associated SitReps are then located through the search function on the NDRRMC website (National Disaster Risk Reduction and Management Council 2024) and data are gathered describing the impact types listed above and the provinces they occurred in.

### Tropical cyclone categories

Data describing the TC intensity category was determined from International Best Track Archive (IBTrACS) data using the wind speed at landfall or the closest distance to land (Knapp et al. 2010, 2018). The category was assigned according to the classification outlined by the Japan Meteorological Agency (JMA) (Japan Meteorological Agency 2024), assigning each TC one of the following categories, in order of increasing intensity:

- **Tropical Depression**: wind speed $< 17$ms$^{-1}$
- **Tropical Storm**: 17ms$^{-1}$ $\ge$ wind speed $< 25$ms$^{-1}$
- **Severe Tropical Storm**: 25ms$^{-1}$ $\ge$ wind speed $< 33$ms$^{-1}$
- **Strong Typhoon**: 33ms$^{-1}$ $\ge$ wind speed $< 44$ms$^{-1}$
- **Very Strong Typhoon**: 44ms$^{-1}$ $\ge$ wind speed $< 54$ms$^{-1}$
- **Violent Typhoon**: wind speed $\ge$ 54ms$^{-1}$

Some lower-intensity TCs in the dataset could not be identified within the IBTrACS records, which is expected given different agencies’ reporting on Tropical Depressions are not expected to be uniform within the dataset (National Centers for Environmental Information (NCEI) 2021). For these TCs, their SitReps were used to confirm their classification as Tropical Depressions.

### Criteria and conventions

Impact data are recorded for TC events and their associated SitReps which meet the following criteria:

- TC is classified as a Tropical Depression or stronger;
- SitRep documents the impact of a single TC event (rather than aggregating the impacts of multiple hazards together, for example the impacts of both a TC and the Southwest Monsoon);
- SitRep reported at least one of the following impact types: deaths, affected population, houses damaged, houses destroyed;
- Reported impacts were spatially disaggregated into provinces.

A list of the TCs that are included in the dataset and links for the corresponding SitReps are provided in the Supplementary Material (Sect. 1). Occasionally the SitRep for a TC identified in non-NDRRMC sources could not be identified on the NDRRMC website. It was assumed that the SitRep did not exist for these events and as such they were excluded from the study. This decision reflects the authors’ goal of avoiding bias where possible, since including impact data from alternative sources risks introducing bias between the sources (Gall et al. 2009). In total, 10 SitReps could not be located or did not contain reports of the relevant impact types. A further 15 SitReps reported the impacts of multiple hazards together and were therefore not included in the dataset.

The following conventions were followed during the data collection process:

- The configuration of provinces defined by the Philippine Standard Geographic Codes (PSGC) (Philippine Statistics Authority 2024) was used throughout. The PSGC for 2024 separates the province of Maguindanao into Maguindanao del Norte and Maguindanao del Sur, however these are considered together in this study. Although it is not a province, we also consider the National Capital Region as a separate geographical unit due to its high population and hence its potential for experiencing high impacts;
- Each TC is referred to by its international name, except when the international name is repeated within the study period, in which case the local name is used for one of the instances;
- In the instance where a TC name is repeated and an alternative name does not exist, the second instance of the TC name is given the suffix _2.

The single geographical convention detailed above was followed to avoid geographical bias and retain the ability to directly compare the locations of impacts between events and through time (Gall et al. 2009). Where reported impacts did not match the geographical boundary convention followed in this study, they were allocated against the province that they were geographically situated within. If the reported impacts for a province included only one or a subset of the impact types, it was assumed that the impact for remaining impact type(s) was zero. Similarly, if a province was not mentioned against any of the impact types within a SitRep, it was assumed that it was not directly affected by the TC and was not included in the dataset.

It is important to acknowledge that although steps were taken during the data curation to avoid introducing bias into the impact data, it is possible that additional biases exist within the data source, for example missing data or biases between the data for different impact types, and these are challenging to assess due to a lack of single ground-truth data source.

## Results

### Evaluating impact data coverage

#### Impact magnitudes

The final dataset includes impact data for 85 TC events that occurred between 2010 and 2020 (inclusive). The time series length was chosen to build upon previous studies of TC impact data in the Philippines which either cover shorter time periods (e.g. Santos 2021; Lagmay and Racoma 2019; Yonson et al. 2018; Esteban et al. 2013) or do not consider multiple impact types (e.g. Healey et al. 2023; Gray et al. 2022; Cinco et al. 2016), whilst maintaining a manageable dataset size for manual curation. Over the whole study period, the dataset accounts for nearly 10,000 deaths, over 80 million people affected and over 5 million houses damaged or destroyed, demonstrating the huge significance of TCs on citizens in the Philippines. Reported economic losses from the TC events totalled over 268 and 91 billion PhP for agriculture and infrastructure losses respectively.

**Fig. 1:**
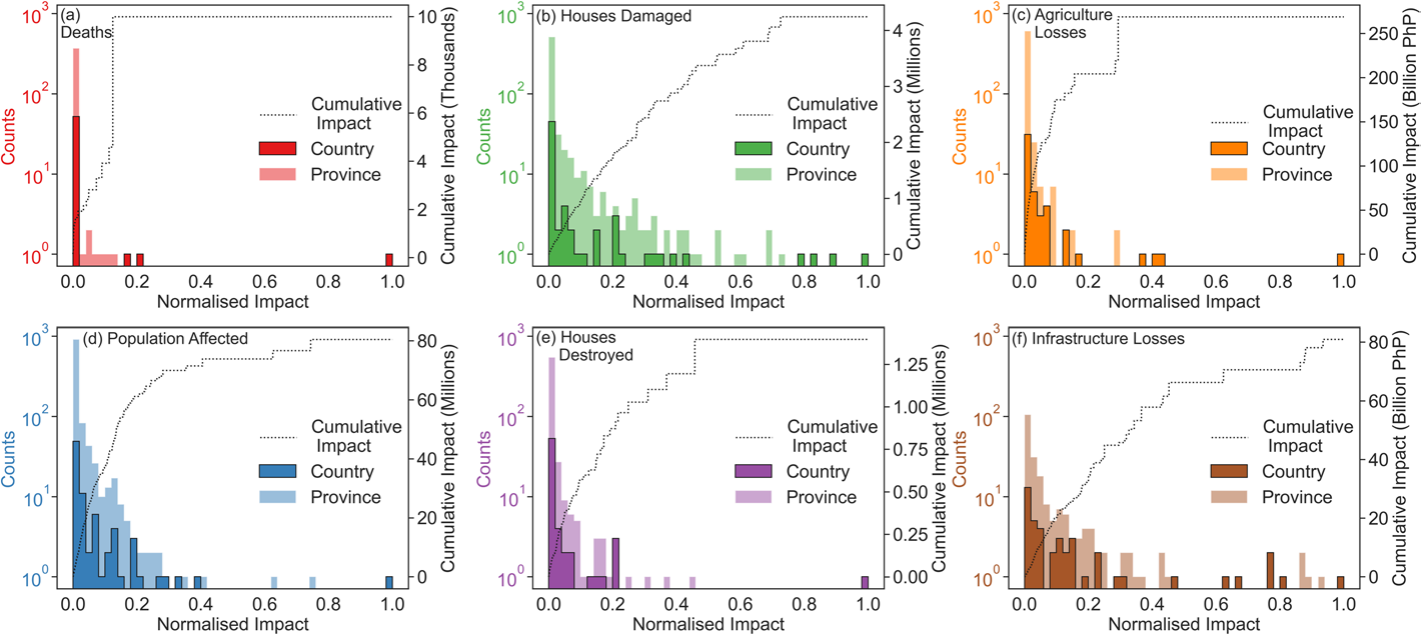
Histograms showing the distributions of min-max normalised TC impacts across the full dataset at country-resolution compared to province-resolution for deaths (**a**), houses damaged (**b**), agricultural economic damage (**c**), population affected (**d**), houses destroyed (**e**), and infrastructural economic damage (**f**). Black dotted lines show step plots of the total cumulative sum of the province-level impacts as normalised impact magnitude increases

The NDRRMC applies a threshold to determine whether an event’s impacts will be published, which includes at least 10 deaths, 50 families or 250 people affected, 50 houses damaged or 5 million PhP of economic damage (National Disaster Risk Reduction and Management Operations Center 2021). Although impact thresholds can prevent small-scale disasters from being represented in an impact dataset, a benefit of spatially disaggregated impact data is that thresholds only apply to the total impact of each TC and not the impacts within its sub-national reporting units (provinces in this case). Here, provinces with low impact values are represented in the dataset and contribute to our understanding of lower-impact TC events in the Philippines. Figure 1 shows that TC impacts in the Philippines are dominated by low values, particularly at the province level, demonstrated by the strong positive skew of the data, suggesting that lower-impact events are a significant component of province-level TC risk in the Philippines. Following a similar approach to Brennan and Danielak (2022), if we assume that the NDRRMC thresholds indicate “low” impacts, these would account for 88% of province-level reports of death tolls, 22% of reports of population affected and 60% of reports of housing damage or destruction. Such instances of lower-impact province-level events can be analysed in further detail to reveal elements of vulnerability that could otherwise be overlooked in aggregated data. The cumulative impact step curves in Fig. 1 also show the significant contribution of lower-impact events to the total impact of all TCs in the dataset, with steeper increases in cumulative impacts at low impact values compared to high. This suggests that over time, the cumulative impacts of frequent, lower-impact TC events become comparative to those of rare, high-impact TCs, making them an important component of impact datasets for DRR research.

Comparing the distributions of province- and country-level impacts in Fig. 1, which have each been min-max normalised between 0 and 1 to allow for comparison, shows that impact data that is spatially disaggregated between the provinces not only provides a larger number of data samples, but has greater coverage across impact magnitudes. A greater range of TC impact outcomes are therefore reflected in the province-resolution data than the sparser country-level impacts, providing a more complete view of overall risk. The representation of low-impact events and higher coverage of impact magnitudes in the dataset therefore makes it particularly valuable for DRR research applications which benefit from a more complete dataset of possible TC events and their corresponding impacts.

#### Spatial resolution

**Fig. 2:**
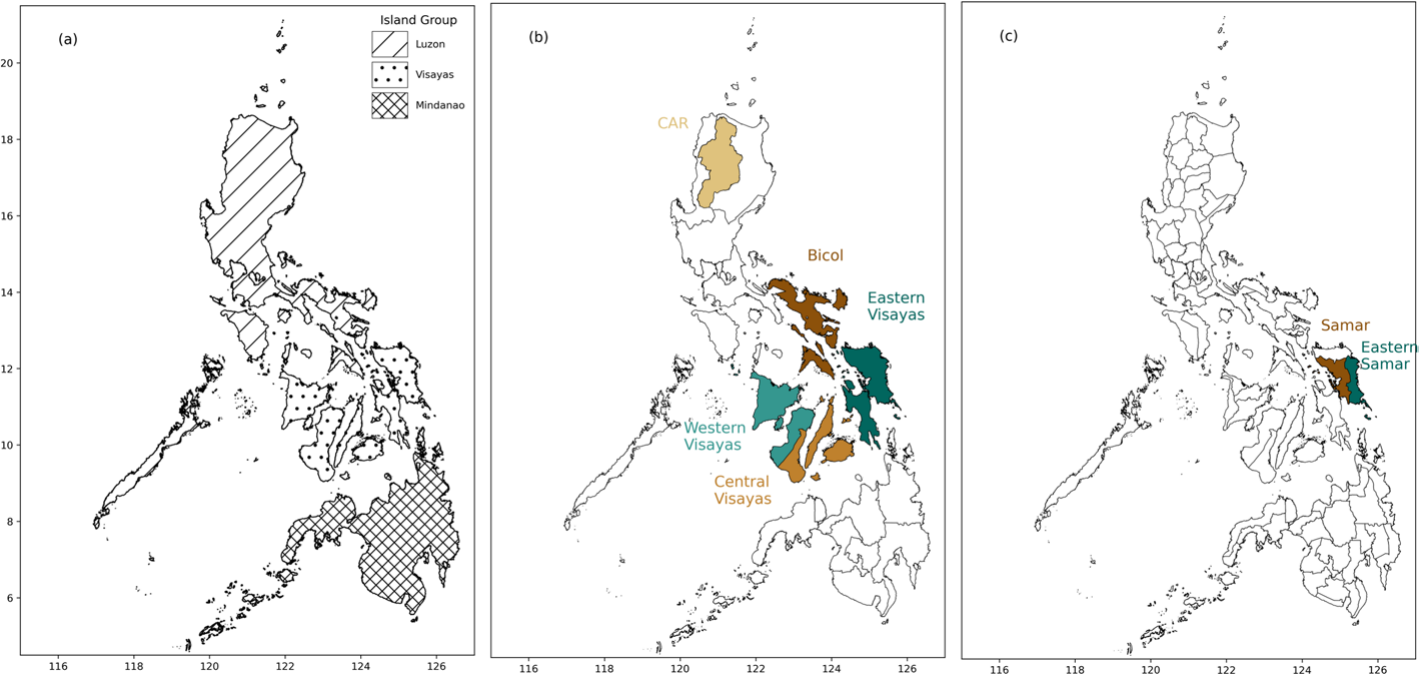
Maps showing administrative divisions of the Philippines for island groups (**a**), regions (**b**), and provinces (**c**), labelled with key locations mentioned in the text

Multiple levels of administrative divisions of the Philippines have been used to show the effect of increasing the resolution of impact data on the understanding of TC risk it provides. To do this, we compare spatial distributions of impact data aggregated to island groups and regions to the higher province-level resolution. Figure 2 shows the island groups, regions, and provinces in the Philippines, labelled with any locations mentioned in the text.

**Fig. 3:**
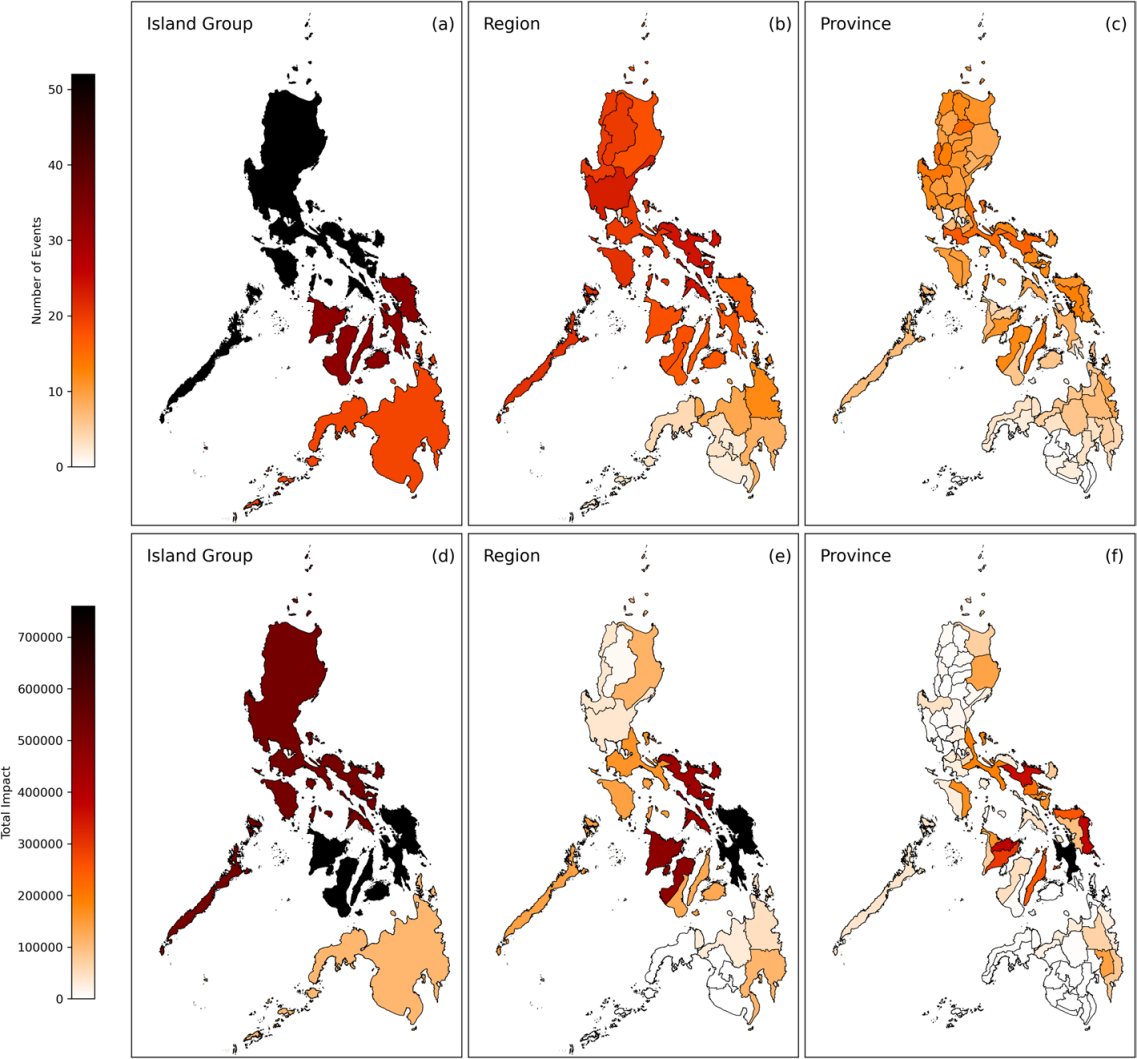
Maps showing the total number of TC events which reported houses destroyed (**a**–**c**) and the total aggregated impact of all TC events which reported houses destroyed (**d**–**f**) within each administrative division for island groups, regions and provinces

Figure 3 demonstrates the additional detail and understanding of TC risk that can be gained by increasing the resolution of the impact data. Figure 3(d-f) shows the total number of houses destroyed over the whole study period for each administrative division. Comparing the spatial distributions of total impacts between island groups (3d), regions (3e), and provinces (3f) reveals finer details and increasing spatial heterogeneity as the resolution increases. It becomes clear that the total aggregated impacts at lower resolutions are dominated by a number of highly impacted provinces which are surrounded by provinces with far lower total impacts. The higher resolution data therefore reveals more detailed patterns in the spatial distribution of TC risk.

**Fig. 4:**
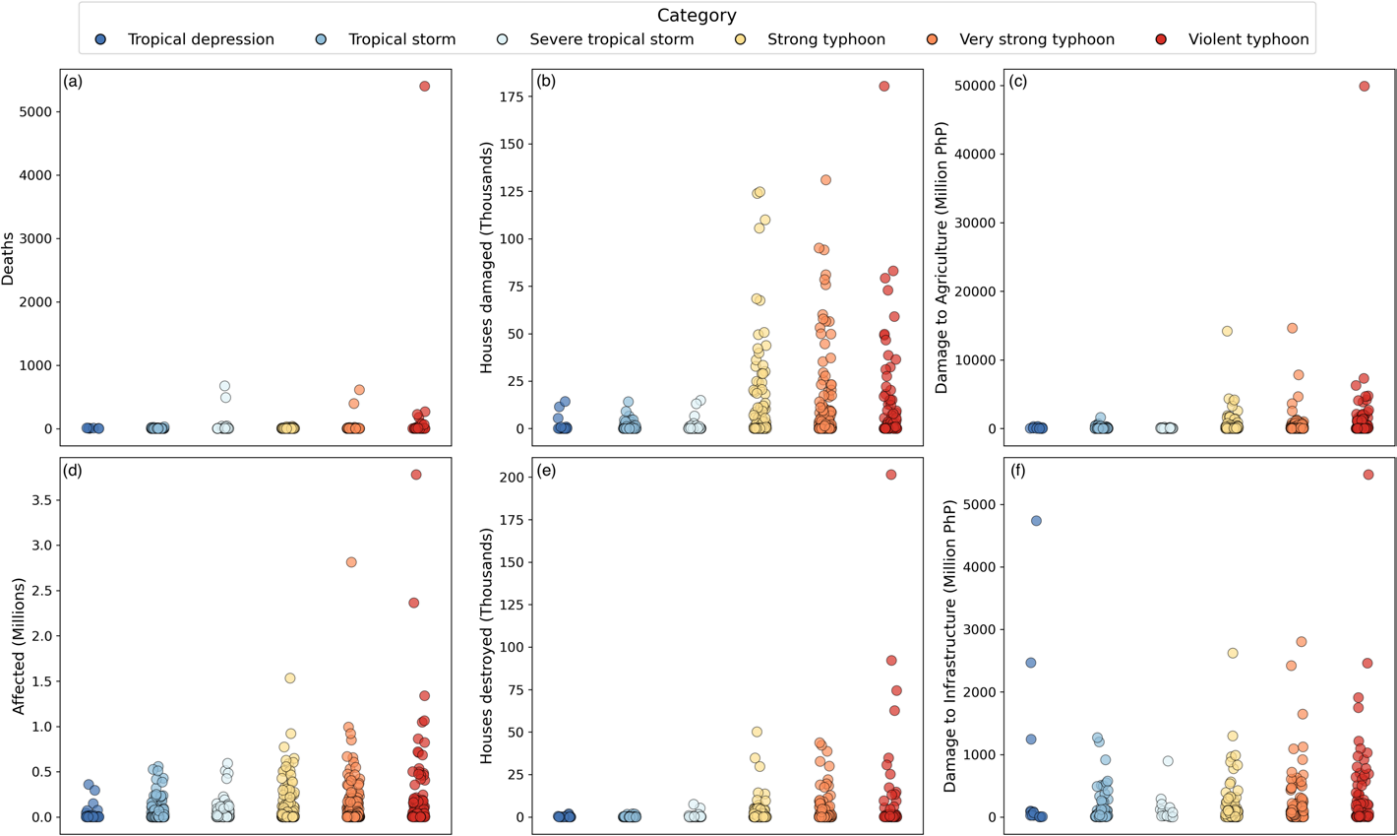
Scatter plot showing the distribution of province-level non-zero impacts for TCs within each category across the dataset for deaths (**a**), houses damaged (**b**), agricultural economic losses (**c**), population affected (**d**), houses destroyed (**e**), and infrastructural economic losses (**f**)

To reveal further characteristics TC risk, the distribution of total impacts can be compared to the number of individual impact reports for housing destruction at each resolution (Fig. 3(a-c)). For example, by examining Figure 3(a) alone, the island group of Luzon appears to have experienced a significantly higher number of events where houses were reported destroyed than the other island groups. However, comparing this to the total number of houses destroyed in each island group (Fig. 3(d)) shows that Visayas experienced a higher total impact despite encountering fewer TC events. This suggests that whilst Luzon is impacted more frequently, Visayas is more vulnerable to housing destruction and Luzon less vulnerable, consistent with previous findings (Baldwin et al. 2023; Healey et al. 2022). Increasing the resolution of the total number of events from island group, to regional, to provincial shows the spatial distribution remains fairly smooth, particularly across Luzon and Visayas (Fig. 3(a–c)). In contrast, the distribution of total impacts reveals more spatial heterogeneity as the resolution is increased (Fig. 3(e & f). For example, within Luzon, the total number of houses destroyed is dominated by the Bicol region, suggesting higher risk to housing destruction compared to the other regions. Conversely, Cordillera Administrative Region (CAR) has the lowest number of houses destroyed across Luzon, despite having a similar number of events reporting impacts, suggesting a lower risk of housing destruction. Similarly, within Visayas the regions of Eastern and Western Visayas contribute much higher numbers of the total houses destroyed than Central Visayas (Fig. 3(e)). As the resolution is further increased to province-level it becomes clear that the majority of the total houses destroyed were situated within just a few provinces, despite the total number of events remaining fairly consistent across provinces (Fig. 3(c & f)). Disparity between bordering provinces’ impacts, for example between Eastern Samar and Samar (Fig. 3(f)), suggests that even when exposed to the same TC events, there are key differences at the province level which can modify the outcome of a TC event. Differences in exposure to TC hazards, for example storm surges, are likely to drive a large part of these, although variability in province-level vulnerability and exposure could also be contributing factors. Spatial features such as these can be seen across the impact types in Figs. 1, 2, 3, 4 and 5 in the Supplementary Material (Sect. 2). The spatially disaggregated data allows differences between the distributions of frequency and magnitude of TC impacts to be identified between island groups, regions and provinces. The examples highlighted here demonstrate the important features of TC risk that can be revealed by increasing the spatial resolution, which are important for understanding vulnerability to TC impacts in DRR research.

#### TC hazard intensity

**Fig. 5:**
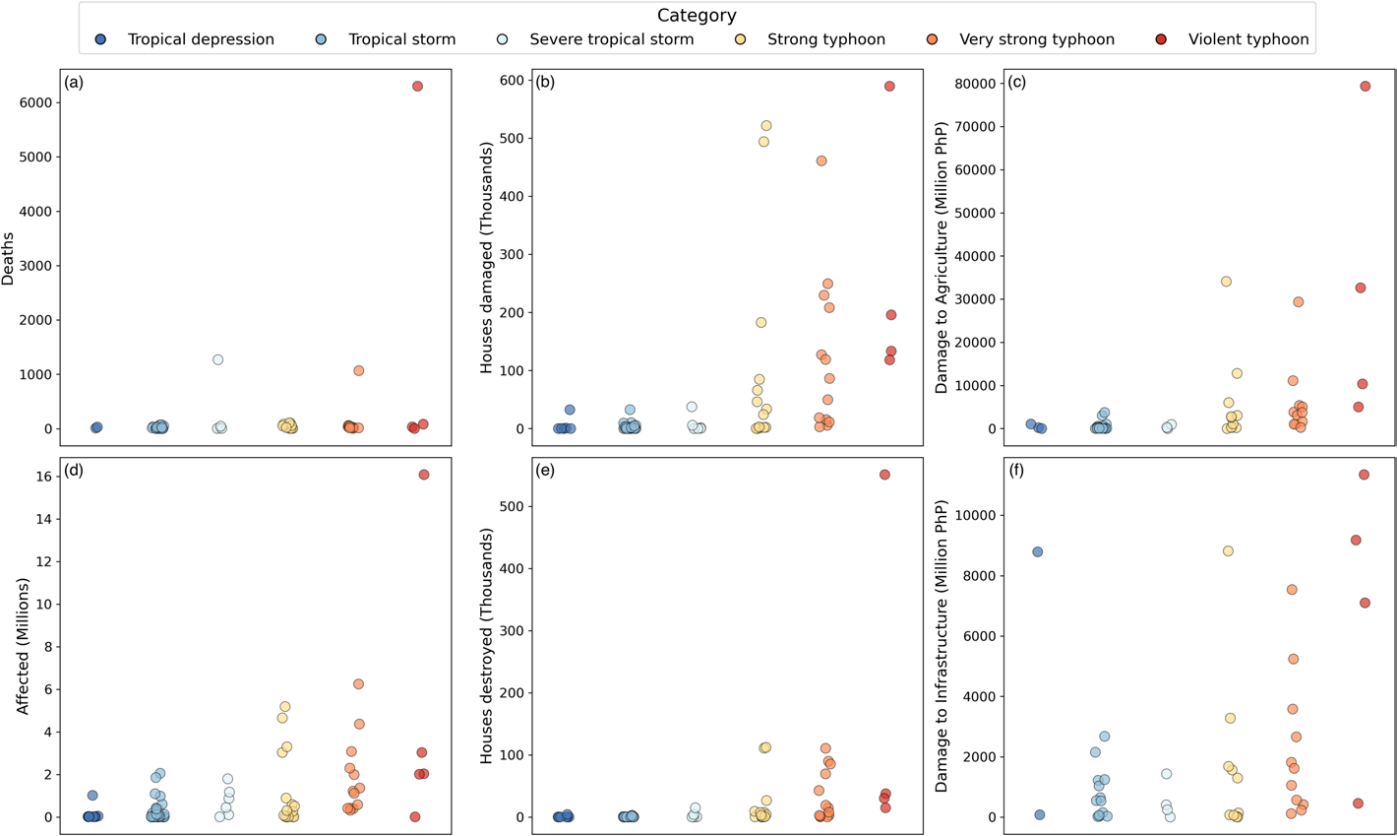
Scatter plot showing the distribution of country-level non-zero impacts for TCs within each category across the dataset for deaths (**a**), houses damaged (**b**), agricultural economic losses (**c**), population affected (**d**), houses destroyed (**e**), and infrastructural economic losses (**f**)

Figure 4 shows the distributions of province-level impacts within each TC intensity category across the full dataset. Most impact types show a general increase in maximum impact and spread of impacts as the TC intensity increases, as expected. The pattern of increasing spread with TC intensity can be partly attributed to the fact that the dataset includes all reported impacts from each TC regardless of affected provinces’ distance from the TC centre. Given that more intense TCs generally have larger radii (Chen et al. 2022; Song et al. 2020), they influence a greater area and impacts include those from provinces which are far from the TC centre where the weather may be less extreme, hence the higher density and spread of impacts for more intense TCs is expected. Further, very intense TCs can cause impacts beyond the region directly affected by the physical TC hazards, for example the displacement of large numbers of people (e.g. UNHCR 2014).

However, there are a number of exceptions to the trend which suggest that TC intensity is not always the dominant factor of impact severity. For example, the distribution of infrastructural economic losses shows a number of high losses attributed to Tropical Depressions (the lowest TC intensity in the dataset), with all intensity categories apart from Violent Typhoons having a lower maximum loss than Tropical Depressions (Fig. 4(f)). Given that the remaining impact types tend to show higher maximum impacts for TCs more intense than Tropical Depressions, suggesting these TCs were more damaging, their impact on infrastructure did not always follow the same pattern. These characteristics indicate that the relationship between province-level infrastructure losses and TC intensity is not necessarily simple, instead suggesting other factors influencing infrastructure vulnerability can also play an important role in TC impacts and are therefore central to understanding TC impact data for DRR applications. One example of this is Tropical Depression Usman (2018), which was responsible for several of the highest infrastructure losses in Fig. 4(f), affected over a million people and damaged or destroyed over 36,000 houses. Despite its low intensity category, Tropical Depression Usman caused widespread and intense rainfall leading to flooding and landslides (UN Office for the Coordination of Humanitarian Affairs 2019). Reports following the event highlighted the importance of early warnings and communication and their effect on TC impact risk (UN Office for the Coordination of Humanitarian Affairs 2019). The overall distributions in Figure 4 and individual case studies such as Tropical Depression Usman suggest a complex, non-linear relationship between TC hazard intensity and impacts. However, it should be noted that the limited size of the impact dataset means that the full variability in risk is unlikely to be captured here.

Notable features also appear in the distributions of houses damaged and destroyed, which show abrupt increases in the maxima and spreads as the intensity category increases from Severe Tropical Storm to Strong Typhoon which could reflect an intensity threshold above which building damage becomes more likely (Fig. 4). This aligns with previous studies on the relationship between wind and building damage, which identify wind speed thresholds and rapid increases in damage with increasing wind speeds (Emanuel 2011; Vickery et al. 2006).

Important insight into TC risk can also be derived from scenarios where high-intensity TCs lead to low impacts, both on the province-level and across the country. Among the distributions of impacts within the most severe TC categories in Fig. 4 are many low province-level impacts. As mentioned, many of these originate from provinces located far enough from the TC centre to avoid the worst TC-related hazards, however this is not always the case. This is seen in Fig. 5, which shows the distributions of country-level impacts aggregated for each TC event within each TC intensity category, revealing some events where extreme impacts were avoided across the country despite the high intensity of the TC. One example is Typhoon Noul (2015), a Violent Typhoon which affected less than 5000 people in total. Reports suggest that early warning and preparation for the storm lessened its impacts on the population (International Federation of Red Cross And Red Crescent Societies 2015). Case studies like these demonstrate the value of an impact dataset with broad spatial and temporal coverage, revealing noteworthy features of TC vulnerability across the spectrum of impact magnitudes and TC intensities.

The increased volume and coverage of the province-level data as well as the inclusion of multiple impact types has enabled a more detailed view of TC risk to develop, including revealing thresholds and relationships between impacts and hazard intensity, as well as revealing “edge cases” which are uncommon but could provide important examples for DRR research.

### Impact type differences

**Fig. 6:**
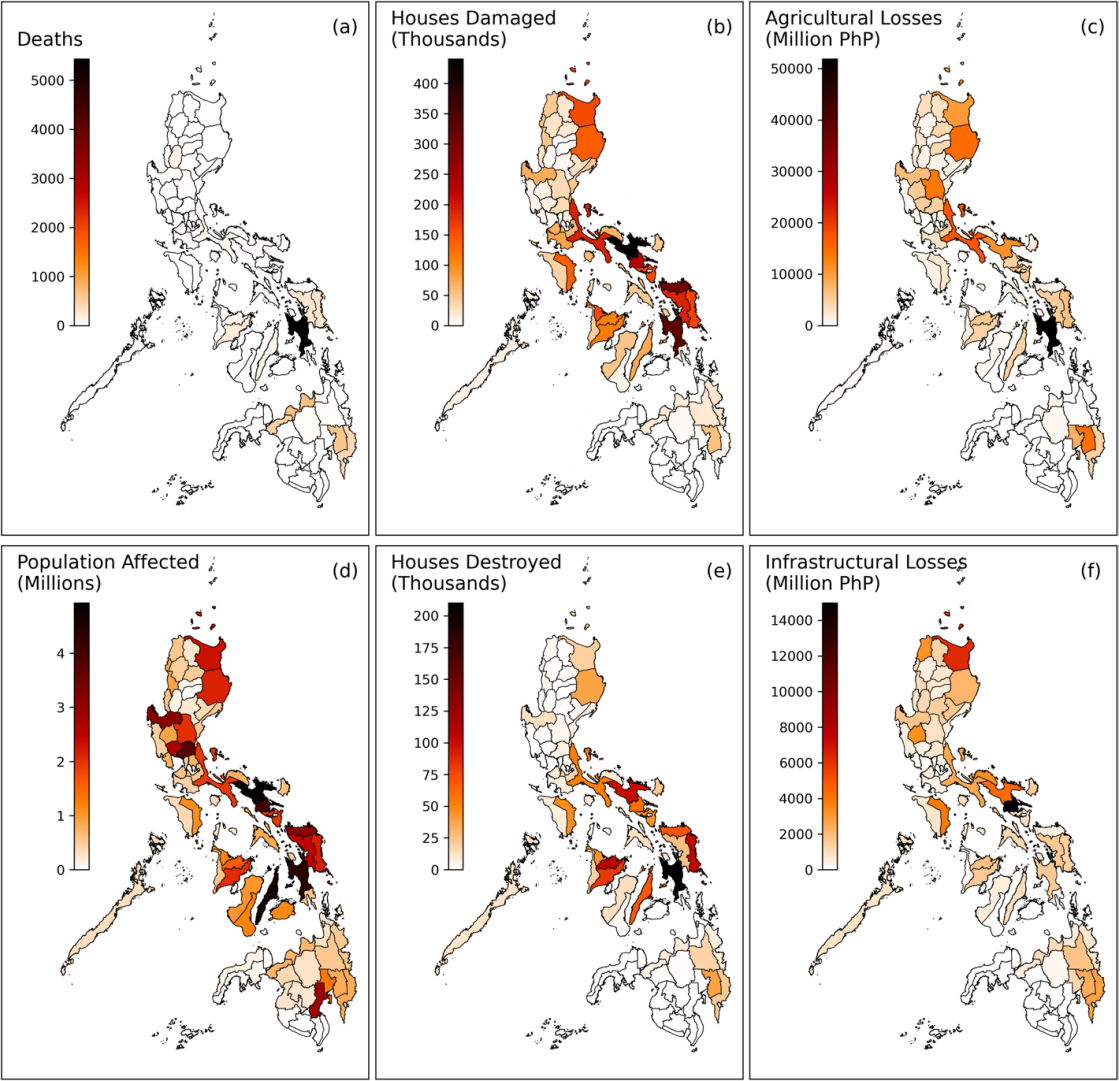
Maps showing the total impact in each province aggregated over the full dataset between 2010 and 2020 for deaths (**a**), houses damaged (**b**), agricultural economic loss (**c**), population affected (**d**), houses destroyed (**e**), and infrastructural economic losses (**f**)

Figure 6 shows the province-level total impact over the whole study period for each of the impact types in the dataset. The significant differences between the spatial patterns of each impact type suggest that differences in province characteristics, as well as the differing behaviour of each impact type, are key features of TC risk in the Philippines. The total number of deaths is dominated by a few provinces within the island groups of Visayas and Mindanao, whilst comparatively few deaths occurred in Luzon (Fig. 6(a)). The same pattern is not seen in the remaining impact types, where several provinces in Luzon feature among the highest impact totals (Fig. 6(b-f). This could be because TC-related deaths over the study period are driven by a few damaging storms in the early part of the study period, including TCs Washi (2011), Bopha (2012) and Haiyan (2013). Whilst Haiyan mainly affected provinces in Visayas, Washi and Bopha caused widespread impacts in the Mindanao region, where vulnerability is high due to poverty and its wider implications (Healey et al. 2023). Some of the remaining highest death tolls are also associated with provinces with high vulnerability. In particular, several high death tolls which occurred after TC Haiyan are attributed to Benguet, a highly mountainous province that suffers frequent landslides (Abancó et al. 2021). This could reflect the unpredictability of landslide-related deaths, which may not benefit as heavily by the improved preparation and warnings after TC Haiyan (Lagmay and Racoma 2019). These case studies show that analysing province-level impacts can indicate different causes of vulnerability in different locations, which is valuable information for many DRR research applications.

The spatial distributions of the remaining impact types are more smooth, but each is dominated by several provinces with high total impacts (Fig. 6(b–f)). Whilst some of the same provinces are among the highest impacted for multiple impact types, there are key differences which could indicate different province-level vulnerabilities to different impact types. For example, high totals of affected population are found throughout the country and are distributed across the island groups, whereas the highest totals within the other impact types are more localised. Comparing the spatial distributions of houses damaged and houses destroyed shows that more provinces across a greater area experienced high numbers of houses damaged, whilst for houses that were completely destroyed, highly impacted provinces were dominated by a few provinces in Visayas. This further supports the findings in Figure 3, which suggests provinces in Luzon are impacted more frequently but generally have less severe impacts. In addition, the regions of high housing destruction fit well with highly vulnerable regions in Healey et al., who create a high-resolution housing vulnerability index using various datasets including socioeconomic indicators and physical housing characteristics (Healey et al. 2022).

High agricultural and infrastructural economic losses exhibit much lower incidence in Visayas compared to other impact types and are dominated mostly by provinces in Luzon (Fig. 6(c & f)). This suggests that economic losses do not always correlate with other types of loss experienced during TCs, such as the loss of homes. Given that economic losses are commonly chosen to represent overall impact severity of disasters, it is important that these differences between the spatial patterns of each impact type, and the vulnerability underlying them, are considered. Differences can also be seen between the distributions of the two types of economic losses, likely reflecting differences in exposure between agricultural and infrastructural assets.

The differences revealed by comparing the spatial patterns of each impact type show that considering multiple types of impact not only supports further understanding of TC vulnerability, but also provides a more holistic view of TC risk than a single impact type, which could introduce biases in DRR research.

### Discussion

Historical impact datasets are pivotal to many DRR applications and can be used as a standalone resource for performing spatial, temporal, and other statistical analyses of impacts to identify patterns in risk and vulnerability. Impact data can also be used alongside other datasets that describe hazard, geographical and socioeconomic features to understand risk and vulnerability further in applications such as risk mapping and impact-based forecasting. Given that these can influence decisions regarding disaster early warning systems and mitigation strategies, it is important to consider features of impact data coverage and quality that can affect the utility of DRR outputs (Wirtz et al. 2014; Kron et al. 2012; Romão and Paupério 2016). For example, the accuracy of impact data must be considered, which is challenging given the inherent uncertainty involved in accounting for disaster losses (Romão and Paupério 2016; Gall et al. 2009). In this study, we verified our dataset against the EM-DAT disaster loss database to ensure that the dataset does not contain major biases in the TC events included. This was important given our aim of capturing a complete time series of TC impacts as far as possible within the scope of the study, with minimal inaccuracy or missingness introduced on the whole. It should however be recognised that accounting for disaster impacts is challenging (Gall et al. 2009). Whilst efforts were made to avoid imposing any additional bias on the impact data, it is important to acknowledge that any biases inherent to the data source could carry through into DRR research based upon the data. Although impact datasets are highly useful indicators of the magnitude of disaster impacts, particularly when comparing between multiple events or locations, DRR researchers should exercise caution when analysing impacts in very high levels of detail.

The communication of disaster risk and its uncertainty is critical in high-risk regions like the Philippines. However, to be effective, research has suggested that disseminated risk information should be specific, contextualised, and probabilistic, which can be particularly difficult for unprecedented events which may be unlike those experienced in the recent past (Yore and Walker 2021; Lagmay and Racoma 2019; de Leon 2019). Historical impact data catalogues represent just a snapshot of possible outcomes and are limited to relatively short time series. Since higher spatial and temporal coverage in impact datasets captures a greater range of hazard and impact magnitudes, more extensive impact datasets are likely to reflect the real-world distribution of disaster events and their impacts more closely, which will in turn be reflected in the DRR outputs grounded in this data. Therefore, choices regarding the temporal and spatial coverage of the curated data reflect the authors’ aim to compile a dataset which improves the understanding it provides into the country’s TC risk. The country-wide, 11-year time series captures events with a wide range of TC intensities and impact magnitudes, as shown in Figs. 1, 4, and 5, which was further increased by leveraging the spatial disaggregation of the impact data. The dataset is therefore useful for representing overall TC risk but can also be interrogated to reveal important aspects of event- or location-specific vulnerability which can inform future DRR practices. The province-level resolution of the impact data and the choice to include all TC impacts, rather than imposing impact thresholds, ensured that the dataset captured instances of both low and high impacts. Analysing the distributions of impact magnitudes showed the importance of small-scale impacts for overall TC impact risk, which could be particularly important in locations which experience frequent, lower-intensity TC events. Not only does higher coverage of impact data increase the number of instances that represent unique circumstances, examples of which were highlighted throughout the results section, it also means the dataset is likely to be closer to the real-world distribution of possible TC impacts, including low-impact events. This means the historical catalogue is more likely to be able to provide analogous examples to future TC events and their impacts, which is crucial given the influence of climate change on TC activity and potential corresponding impacts. However, the length of a historical impact time series is inherently limited and extending the study period and increasing the spatial resolution can only improve the coverage of events by a finite amount. Therefore, future work could involve simulating physically plausible TCs which have different characteristics to those in the historical impact record (e.g. Meiler et al. 2022) or synthesising TCs from climate model outputs to explore the change in TC impacts under climate change (e.g. Delfino et al. 2023). Further, to extend the investigation into low-impact TC events, analysing TC events where impacts do not occur alongside those which cause impacts could further improve our understanding of TC risk and how this can be minimised.

The distribution of TC intensities and their wide-ranging impacts in the dataset demonstrates that the relationship between TC hazards and the resulting impacts is complex (Figs. 4 and 5), supporting findings from previous research (Quesada-Román et al. 2019; Do and Kuleshov 2023; Sarker and Adnan 2024). Understanding this relationship and its intricacies is valuable for DRR applications such as impact forecasting, helping to provide contextualised and probabilistic risk information. As well as analysing low- and high-impact events to understand this relationship further, examining spatial patterns within the dataset reveals features which reflect underlying differences in TC risk, which can be substantial even between bordering locations. Comparing the patterns in impact data disaggregated between island groups, regions and provinces shows that significant spatial heterogeneity is revealed at province-level, highlighting the value of disaggregated, high-resolution impact data for understanding spatial differences in risk (Fig. 3).

We would expect further variability in impacts to be revealed if the resolution were increased to municipality level (e.g. Healey et al. 2022, 2023; Lloyd et al. 2022; Gray et al. 2022), however the authors were constrained by the manual data collection method. There is also potential to improve the dataset’s value by increasing the time series length to capture more TC events, although temporal biases must be considered for long time series (Gall et al. 2009). More readily available, high-resolution impact data, both within the context of TCs in the Philippines and beyond, could lead to more valuable insights and uses within DRR applications. Remote sensing approaches involving image analyses could be one way to identify physical damage to structures such as buildings at high-resolution (Hoque et al. 2017). However, this approach would not be suitable for impacts which could not be identified using imagery, such as affected populations. Alternative studies have explored social sensing methods for estimating disaster impacts, such as using social media data, however, these methods carry their own potential biases which must be considered (Houston et al. 2015). Combining impact data from multiple sources could provide more robust impact data, for example by reducing the potential for missing data, but the biases within and between data sources must be a key consideration.

Leveraging other available datasets which represent different features of disaster risk could also provide opportunities for using the curated impact dataset in DRR research. For example, combining historical impact data with datasets used for TC risk assessments (e.g. Quesada-Román et al. 2022; Quesada-Román and Villalobos-Chacón 2020; Cadiz 2018) could help assess the risk of impacts from future TCs. Data-driven impact modelling and impact-based forecasting similarly rely on a synthesis of hazard, exposure, and vulnerability datasets, as well as high-quality impact data (Merz et al. 2020; ESCAP 2021). For TCs, historical hazard data such as reanalysis data is a useful resource for such applications, as are TC forecasts for assessing future risk, but uncertainty and biases in this data should be considered (e.g. Han et al. 2022; Dulac et al. 2024; Heming et al. 2019; Leroux et al. 2018; Titley et al. 2020). For exposure datasets, the quality, availability, and potential data biases vary depending on the data source and impact type considered (e.g. Hoque et al. 2017; Merz et al. 2020; Pittore et al. 2017). Defining vulnerability and measuring it quantitatively presents a further challenge (Adger 2006; Cutter et al. 2003), although previous studies which have created vulnerability indices specific to TCs in the Philippines could be combined with impact data to provide a more detailed understanding of the individual elements of TC risk (e.g. Healey et al. 2023; Lloyd et al. 2022). These indices are developed for specific impact types, and as the results in this study suggest, vulnerability differs between impact types and locations, supporting the use of context-specific vulnerability data.

The inter-province variability captured by the disaggregated impact data also reveals different spatial patterns between each impact type, showing that the selection of impact type has implications for the picture of risk the data presents. This is an important consideration for DRR researchers when selecting data that best reflects the complex, real-world phenomena they are trying to represent. For DRR research which uses impact data of a single type as a proxy for a disaster’s severity, it is important to acknowledge the limitations and potential biases this can introduce in research outputs. Conversely, the differences between the impact types in the curated dataset shown in Fig. 6, even when aggregated over the entire study period, suggest that considering multiple impact types could give a more comprehensive view of risk and allow DRR research outputs to better reflect the breadth of disaster impacts.

We have demonstrated several ways in which the resolution and coverage of impact data can influence our understanding of risk, with higher spatial and temporal coverage providing richer insight in many cases. We have shown how these features within the curated dataset provide the ability to identify patterns in risk which could have implications for decision making as well as helping to locate areas of interest for further DRR studies. One approach which our dataset enables that reduces the burden of data collection would be to use the dataset to identify locations which have shown interesting attributes at province-level resolution and investigate these specific cases further to reveal sub-province trends in impacts. The analysis carried out in this paper has involved all events and provinces within the dataset, highlighting specific examples to demonstrate its value, but opens up many opportunities for future TC impact analyses.

## Supplementary Information

Below is the link to the electronic supplementary material.

Supplementary file 1 (pdf 6469 KB)

## Data Availability

The data in this study can be accessed at the following DOI, which will be made available upon publication: 10.6084/m9.figshare.26799835
