# Supplementary material for: Tropical cyclone impact data in the Philippines: implications for disaster risk research
Source: Nat Hazards (Dordr). 2025 Jun 20;121(13):15275–96. doi: 10.1007/s11069-025-07394-x (PMC12310784; doi:10.1007/s11069-025-07394-x)
Supplement: Supplementary file 1 — (pdf 6469 KB) [file 11069_2025_7394_MOESM1_ESM.pdf]

Supplementary Material for Tropical Cyclone Impact Data in the Philippines: Implications for Disaster Risk Research

Elizabeth G. Galloway[1,2,*], Jennifer L. Catto[2], Chunbo Luo[3], Stefan Siegert[2], and [2]

[1]UKRI Centre for Doctoral Training in Environmental Intelligence: Data Science and AI for Sustainable Futures, University of Exeter, UK
[2]Department of Mathematics and Statistics, University of Exeter, UK
[3]Department of Computer Science, University of Exeter, UK
[*]Corresponding author: egg203@exeter.ac.uk

Contents

1 List of NDRRMC Reports **1**

2 Additional Figures **4**
2.1 Figure 1: Deaths 4
2.2 Figure 2: Affected 5
2.3 Figure 3: Houses Damaged 6
2.4 Figure 4: Agricultural Economic Loss 7
2.5 Figure 5: Infrastructural Economic Loss 8

1 List of NDRRMC Reports

The NDRRMC SitReps referenced below can be accessed via the URLs provided in the citations, or alternatively using the Internet Archive, which contains a record of internet content and can be accessed via: `https://web.archive.org/`, where the SitRep URL can be searched, for example: `https://web.archive.org/web/20250000000000*/https://ndrrmc.gov.ph/attachments/article/2272/FINAL_REPORT_re_Effects_of_Tropical_Storm_AURING_(Sonamu)_03-04JAN2013.pdf`.

1. Conson [1]
2. Megi [2]
3. Aere [3]
4. Songda [4]
5. Dodong [5]
6. Egay [6]
7. Meari [7]
8. Juaning [8]
9. Nanmadol [9]
10. Nesat [10]
11. Nalgae [11]
12. Banyan [12]
13. Washi [13]
14. Ferdie [14]

15. Igme [15]
16. Jelawat [16]
17. Gaemi [17]
18. Son-Tinh [18]
19. Bopha [19]
20. Wukong [20]
21. Sonamu [21]
22. Bising [22]
23. Crising [23]
24. Rumbia [24]
25. Utor [25]
26. Kong-Rey [26]
27. Odette [27]
28. Krosa [28]
29. Haiyan [29]
30. Lingling [30]
31. Kajiki [31]
32. Caloy [32]
33. Rammasun [33]
34. Matmo [34]
35. Luis [35]
36. Fung-Wong [36]
37. Sinlaku [37]
38. Hagupit [38]
39. Jangmi [39]
40. Mekkhala [40]
41. Maysak [41]
42. Noul [42]
43. Linfa [43]
44. Ineng [44]
45. Mujigae [45]
46. Koppu [46]
47. Melor [47]
48. Onyok [48]
49. Nida [49]
50. Meranti [50]
51. Helen [51]

52. Sarika [52]
53. Haima [53]
54. Tokage[54]
55. Nock-Ten [55]
56. Auring [56]
57. Crising_2 [57]
58. Pakhar [58]
59. Doksuri [59]
60. Khanun [60]
61. Damrey [61]
62. Haikui [62]
63. Kirogi [63]
64. Kai-Tak [64]
65. Tembin [65]
66. Bolaven [66]
67. Sanba [67]
68. Mangkhut [68]
69. Yutu [69]
70. Usagi [70]
71. Usman [71]
72. Amang [72]
73. Chedeng [73]
74. Danas [74]
75. Marilyn [75]
76. Kalmaegi [76]
77. Kammuri [77]
78. Phanfone [78]
79. Vongfong [79]
80. Ofel [80]
81. Saudel [81]
82. Molave [82]
83. Goni [83]
84. Vamco [84]
85. Krovanh [85]

2 Additional Figures

2.1 Figure 1: Deaths

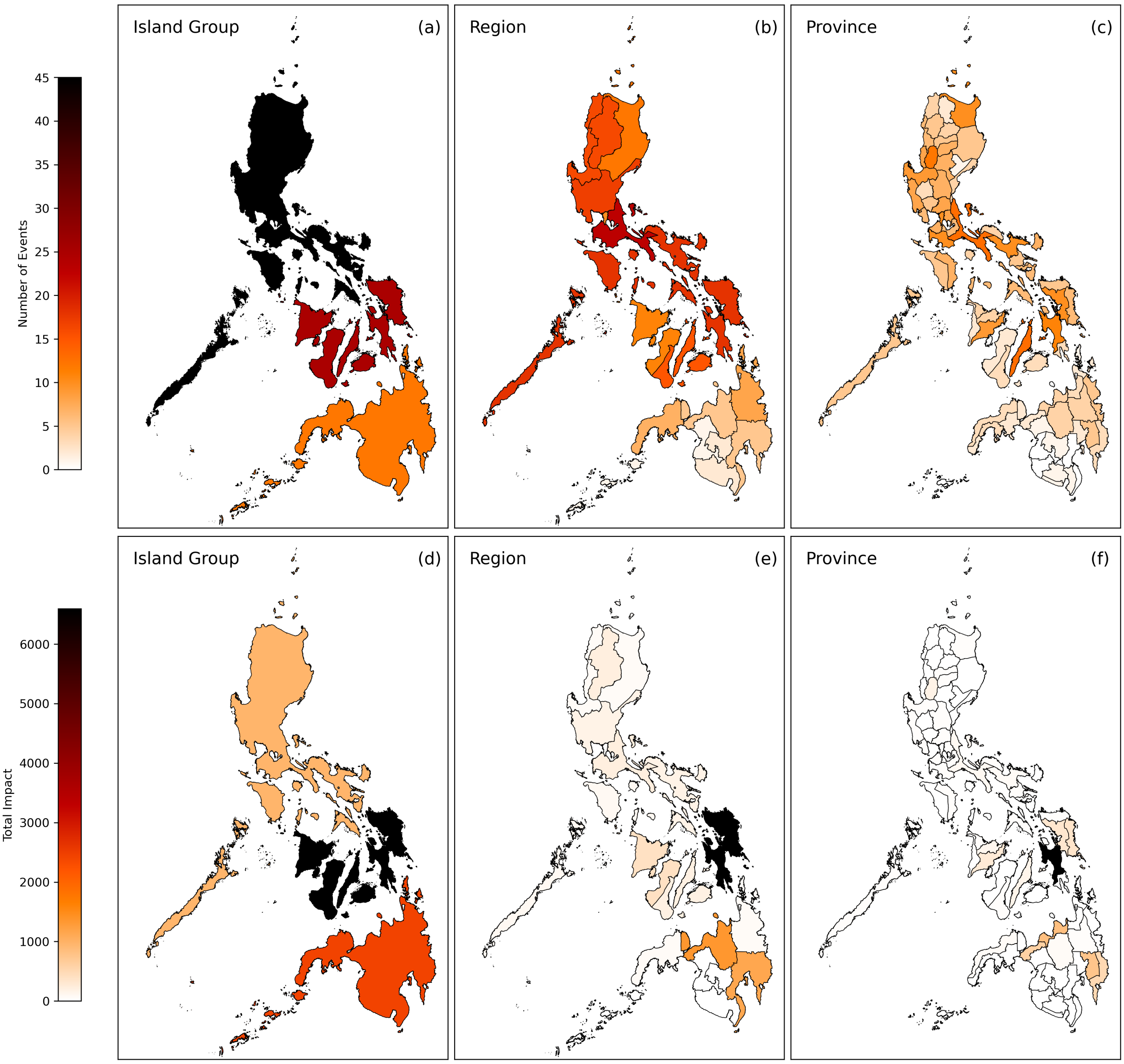

Figure 1: Maps showing the total number of TC events which reported deaths (a-c) and the total aggregated impact of all TC events which reported deaths (d-f) within each administrative division for island groups, regions and provinces.

2.2 Figure 2: Affected

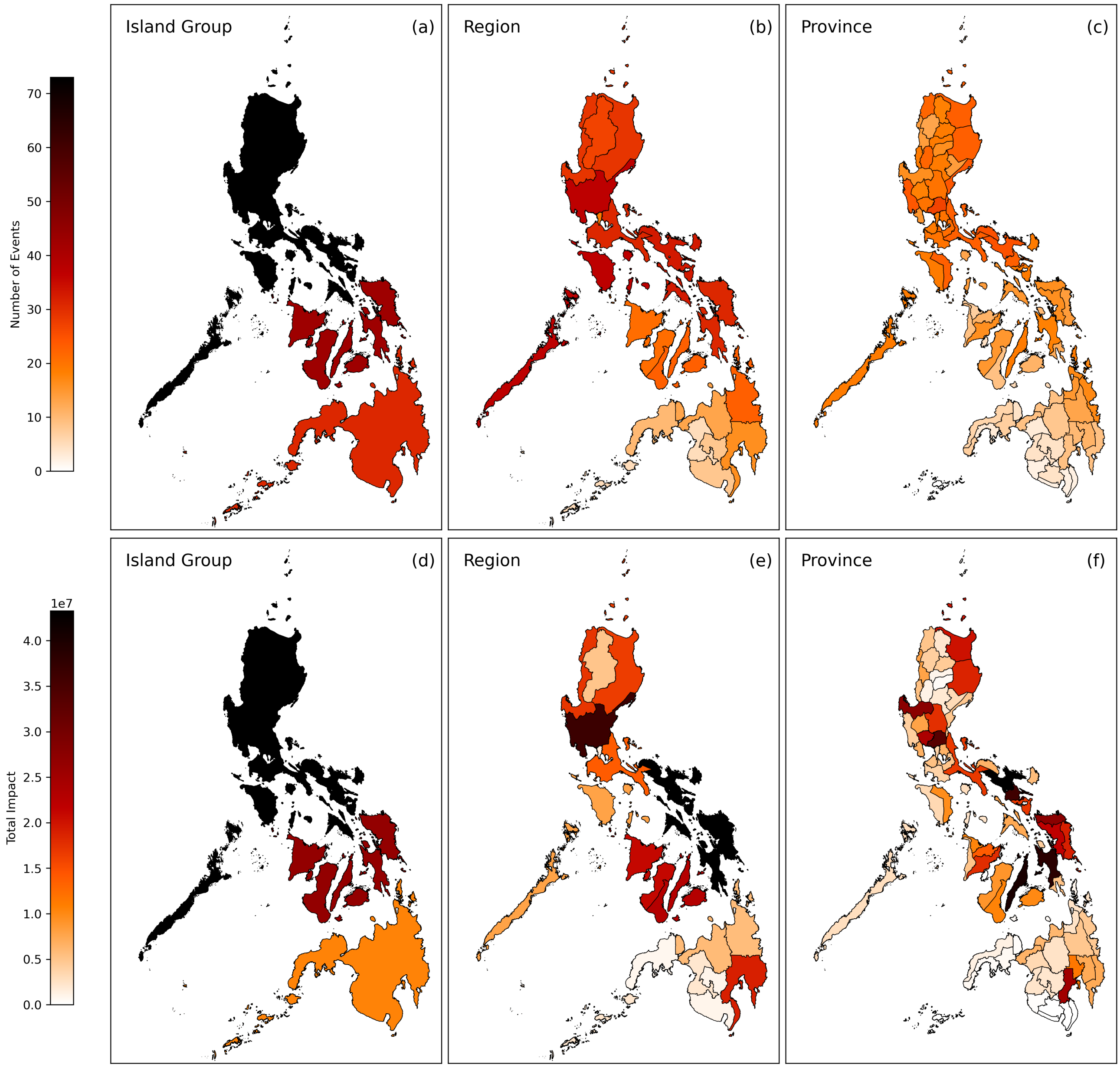

Figure 2: Maps showing the total number of TC events which reported affected populations (a-c) and the total aggregated impact of all TC events which reported affected populations (d-f) within each administrative division for island groups, regions and provinces.

2.3 Figure 3: Houses Damaged

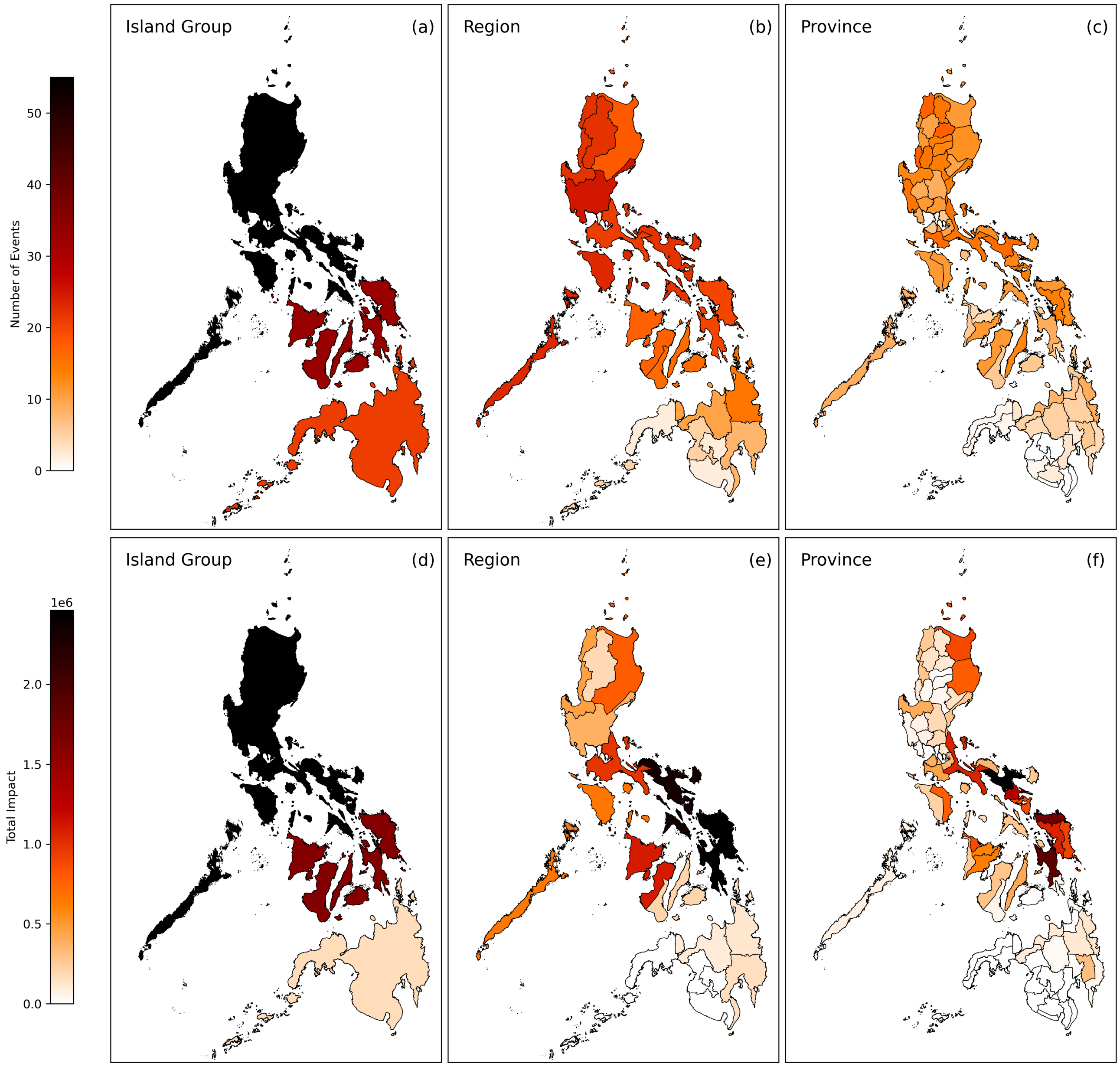

Figure 3: Maps showing the total number of TC events which reported houses damaged (a-c) and the total aggregated impact of all TC events which reported houses damaged (d-f) within each administrative division for island groups, regions and provinces.

2.4 Figure 4: Agricultural Economic Loss

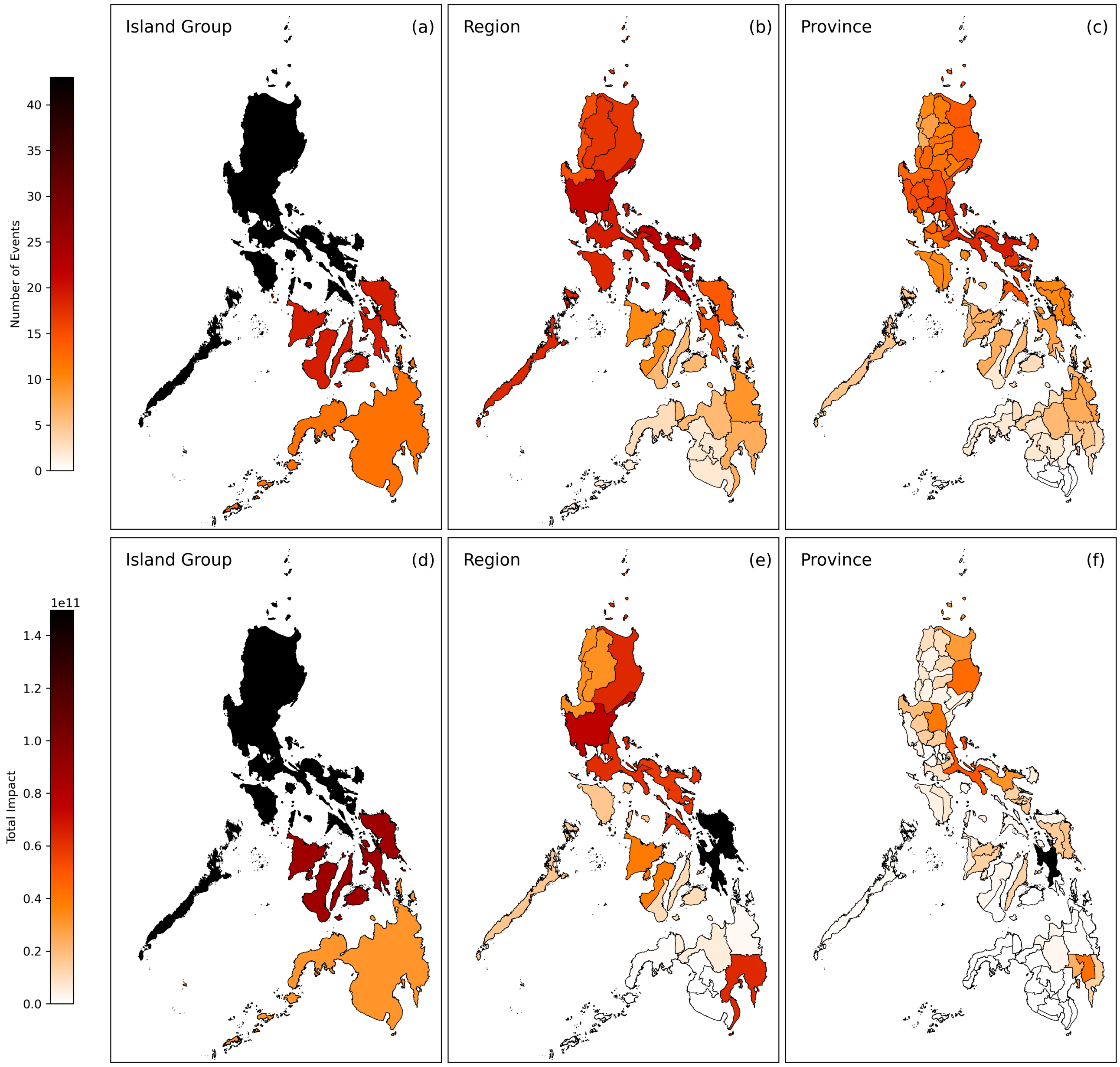

Figure 4: Maps showing the total number of TC events which reported agricultural losses (a-c) and the total aggregated impact of all TC events which reported agricultural losses (d-f) within each administrative division for island groups, regions and provinces.

2.5 Figure 5: Infrastructural Economic Loss

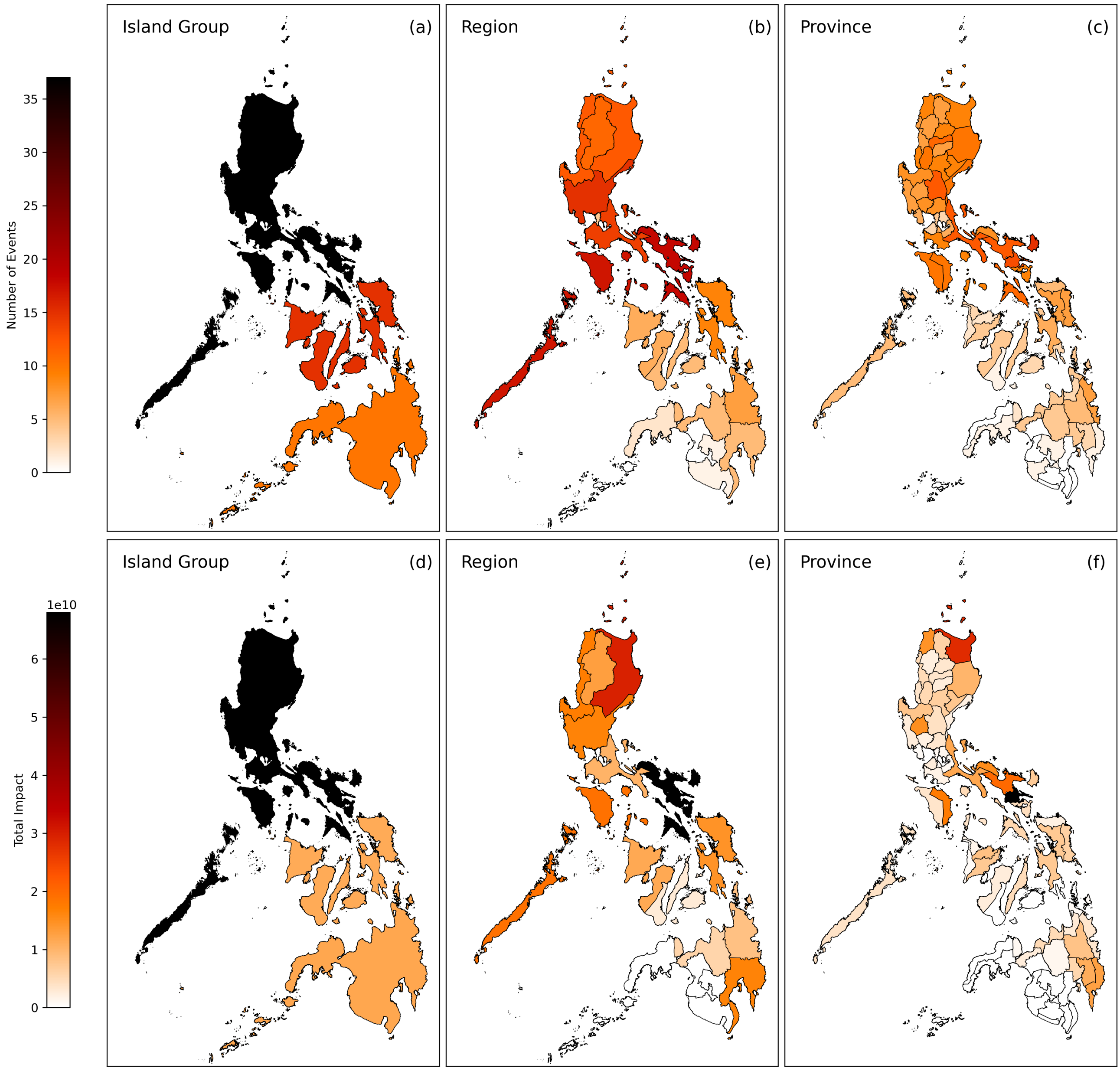

Figure 5: Maps showing the total number of TC events which reported infrastructural losses (a-c) and the total aggregated impact of all TC events which reported infrastructural losses (d-f) within each administrative division for island groups, regions and provinces.

References

[1] NDRRMC, "NDRRMC SitRep for TC Conson," 2010. [Online]. Available: https://ndrrmc.gov.ph/attachments/article/4158/Final_Report_on_the_Effects_of_TS_Basyang_CONSON_GlideNo_TC-2010-000131-PHL.pdf

[2] ——, "NDRRMC SitRep for TC Megi," 2010. [Online]. Available: https://ndrrmc.gov.ph/attachments/article/1554/SitRep_No_28_re_Typhoon_Juan_as_of_30OCT2010.pdf

[3] ——, "NDRRMC SitRep for TC Aere," 2011. [Online]. Available: https://ndrrmc.gov.ph/attachments/article/1656/Final_Report_on_Tropical_Storm_BEBENG_AERE_8-11MAY2011.pdf

[4] ——, "NDRRMC SitRep for TC Songda," 2011. [Online]. Available: https://ndrrmc.gov.ph/attachments/article/1662/Final_Report_re_Preparedness_Initiatives_and_Effects_of_Typhoon_CHEDENG_SONGDA_as_of_20to28MAY2011.pdf

[5] ——, "NDRRMC SitRep for TC Dodong," 2011. [Online]. Available: https://ndrrmc.gov.ph/attachments/article/4160/Final_Report_Tropical_Storm_DODONG.pdf

[6] ——, "NDRRMC SitRep for TC Egay," 2011. [Online]. Available: https://ndrrmc.gov.ph/attachments/article/1676/SitRep_No_08_re_Tropical_Depression_EGAY_as_of_22JUN2011_0800H.pdf

[7] ——, "Ndrrmc sitrep for tc meari."

[8] ——, "NDRRMC SitRep for TC Juaning," 2011. [Online]. Available: https://ndrrmc.gov.ph/attachments/article/1696/SitRep_No_14_for_Tropical_Storm_JUANING_as_of_2AUG2011_0600H.pdf

[9] ——, "NDRRMC SitRep for TC Nanmadol," 2011. [Online]. Available: https://ndrrmc.gov.ph/attachments/article/4160/Final_Report_re_Effects_of_Typhoon_MINA_21to29Aug2011.pdf

[10] ——, "NDRRMC SitRep for TC Nesat," 2011. [Online]. Available: https://ndrrmc.gov.ph/attachments/article/1748/SitREp_No_26_re_effects_of_Typhoon_PEDRING_as_of_11OCT2011_0600H.pdf

[11] ——, "NDRRMC SitRep for TC Nalgae," 2011. [Online]. Available: https://ndrrmc.gov.ph/attachments/article/1763/SitRep_no_13_Effects_of_Typhoon_QUIEL_as_of_11OCT2011_0600H.pdf

[12] ——, "NDRRMC SitRep for TC Banyan," 2011. [Online]. Available: https://ndrrmc.gov.ph/attachments/article/1774/SitRep_No_14_re_Effects_of_tropical_Storm_RAMON_as_of_16OCT2011_0600H.pdf

[13] ——, "NDRRMC SitRep for TC Washi," 2012. [Online]. Available: https://ndrrmc.gov.ph/attachments/article/1809/SitRep_No_47_re_effects_of_tropical_Storm_SENDONG_and_Status_of_Emergency_Response_Operations_as_of_26JAN2012_0800H.pdf

[14] ——, "NDRRMC SitRep for TC Ferdie," 2012. [Online]. Available: https://ndrrmc.gov.ph/attachments/article/2034/Final_Report_re_Effects-of_Tropical_Depression_FERDIE_and_Emergency_Response_Management_as_of_20SEP2012.pdf

[15] ——, "NDRRMC SitRep for TC Igme," 2012. [Online]. Available: https://ndrrmc.gov.ph/attachments/article/2060/SitRep_No_14_re_Preparedness_and_Response_to_the_Effects_of_Tropical_Storm_IGME_as_of_30AUG2012_0600H.pdf

[16] ——, "NDRRMC SitRep for TC Jelawat," 2012. [Online]. Available: https://ndrrmc.gov.ph/attachments/article/2120/SitRep_No_19_Effects_of_Typhoon_LAWIN_as_of_10OCT2012.pdf

[17] ——, "NDRRMC SitRep for TC Gaemi," 2012. [Online]. Available: https://ndrrmc.gov.ph/attachments/article/2136/SitRep_No_06_Effects_of_Tropical_Storm_MARCE_as_of_06OCT2012_0600H.PDF

[18] ——, "NDRRMC SitRep for TC Son-Tinh," 2012. [Online]. Available: https://ndrrmc.gov.ph/attachments/article/2174/SitRep_No_17_Effects_of_Tropical_Storm_OFEL_as_of_01NOV2012_0600H.pdf

[19] ——, "NDRRMC SitRep for TC Bopha," 2012. [Online]. Available: https://ndrrmc.gov.ph/attachments/article/2245/SitRep_No_38_Effects_of_Typhoon_PABLO_as_of_25DEC2012_0600H.pdf

[20] ——, "NDRRMC SitRep for TC Wukong," 2013. [Online]. Available: https://ndrrmc.gov.ph/attachments/article/2259/SitRep_No_13_Effects_and_Response_for_Tropical_Depression_QUINTA_as_of_05JAN2013_0600H.pdf

[21] ——, "NDRRMC SitRep for TC Sonamu," 2013. [Online]. Available: https://ndrrmc.gov.ph/attachments/article/2272/FINAL_REPORT_re_Effects_of_Tropical_Storm_AURING_(Sonamu)_03-04JAN2013.pdf

[22] ——, "NDRRMC SitRep for TC Bising," 2013. [Online]. Available: https://ndrrmc.gov.ph/attachments/article/2283/FINAL_REPORT_re_Effects_of_Tropical_Depression_BISING_11_to_13JAN2015.pdf

[23] ——, "NDRRMC SitRep for TC Crising," 2013. [Online]. Available: https://ndrrmc.gov.ph/attachments/article/2657/FINAL_REPORT_re_Effects_and_RESPONSE_for_TROPICAL_DEPRESSION_CRISING_(Shanshan)_18-21FEB2013.pdf

[24] ——, "NDRRMC SitRep for TC Rumbia," 2013. [Online]. Available: https://ndrrmc.gov.ph/attachments/category/12/FINAL_REPORT_re_Effects_of_Tropical_Storm_GORIO_RUMBIA_27JUN_to_01JUL2013.pdf

[25] ——, "NDRRMC SitRep for TC Utor," 2013. [Online]. Available: https://ndrrmc.gov.ph/attachments/category/12/FINAL_REPORT_re_Effects_and_Emergency_Management_of_Typhoon_LABUYO_UTOR_09-12AUG2013.pdf

[26] ——, "NDRRMC SitRep for TC Kong-Rey," 2013. [Online]. Available: https://ndrrmc.gov.ph/attachments/article/2657/FINAL_REPORT_re_Preparedness_Measures_and_Effects_of_Tropical_Storm_NANDO_(KONG-REY)_25-29AUG2013.pdf

[27] ——, "NDRRMC SitRep for TC Odette," 2013. [Online]. Available: https://ndrrmc.gov.ph/attachments/article/2657/FINAL_REPORT_re_Effects_of_Typhoon_ODETTE_(USAGI)_16-22SEP2013.pdf

[28] ——, "NDRRMC SitRep for TC Krosa," 2013. [Online]. Available: https://ndrrmc.gov.ph/attachments/article/2657/FINAL_REPORT_re_Effects_of_Typhoon_VINTA_KROSA_29OCT-01NOV2013.pdf

[29] ——, "NDRRMC SitRep for TC Haiyan," 2013. [Online]. Available: https://ndrrmc.gov.ph/attachments/article/1329/FINAL_REPORT_re_Effects_of_Typhoon_YOLANDA_(HAIYAN)_06-09NOV2013.pdf

[30] ——, "NDRRMC SitRep for TC Lingling," 2014. [Online]. Available: https://ndrrmc.gov.ph/attachments/article/2783/FINAL_REPORT_re_Effects_of_Tropical_Depression_AGATON_17_-_20JAN2014.pdf

[31] ——, "NDRRMC SitRep for TC Kajiki," 2014. [Online]. Available: https://ndrrmc.gov.ph/attachments/article/2785/TS_BASYANG_2014.pdf

[32] ——, "NDRRMC SitRep for TC Caloy," 2014. [Online]. Available: https://ndrrmc.gov.ph/attachments/article/2785/TD_CALOY_2014.pdf

[33] ——, "NDRRMC SitRep for TC Rammasun," 2014. [Online]. Available: https://ndrrmc.gov.ph/attachments/article/1293/Effects_of_Typhoon_Glenda_(RAMMASUN)_Final_Report_16SEP2014.pdf

[34] ——, "NDRRMC SitRep for TC Matmo," 2014. [Online]. Available: https://ndrrmc.gov.ph/attachments/article/2785/TY_HENRY_2014.pdf

[35] ——, "NDRRMC SitRep for TC Luis," 2014. [Online]. Available: https://ndrrmc.gov.ph/attachments/category/12/FINAL_REPORT_re_Effects_of_Typhoon_LUIS_KALMAEGI_12_-_15SEP2014.pdf

[36] ——, "NDRRMC SitRep for TC Fung-Wong," 2014. [Online]. Available: https://ndrrmc.gov.ph/attachments/article/2785/TS_MARIO_2014.pdf

[37] ——, "NDRRMC SitRep for TC Sinlaku," 2014. [Online]. Available: https://ndrrmc.gov.ph/attachments/article/2785/TS_QUEENIE_2014.pdf

[38] ——, "NDRRMC SitRep for TC Hagupit," 2014. [Online]. Available: https://ndrrmc.gov.ph/attachments/article/4030/FINAL_REPORT_re_Effects_of_Typhoon_RUBY_HAGUPIT_04_10DEC2014.pdf

[39] ——, "NDRRMC SitRep for TC Jangmi," 2014. [Online]. Available: https://ndrrmc.gov.ph/attachments/article/2785/TS_SENIANG_2014.pdf

[40] ——, "NDRRMC SitRep for TC Mekkhala," 2015. [Online]. Available: https://ndrrmc.gov.ph/attachments/article/1379/SitRep_No_10_re_Effects_of_Tropical_Storm_Amang_MEKKHALA_issued_on_20JAN2015_1800H.pdf

[41] ——, "NDRRMC SitRep for TC Maysak," 2015. [Online]. Available: https://ndrrmc.gov.ph/attachments/article/1421/FINAL_REPORT_re_Preparedness_Measures_and_Effects_of_Typhoon_CHEDENG_MAYSAK_01_-_05APR2015.pdf

[42] ——, "NDRRMC SitRep for TC Noul," 2015. [Online]. Available: https://ndrrmc.gov.ph/attachments/article/1441/FINAL_REPORT_re_Preparedness_Measures_and_Effects_of_Typhoon_DODONG_NOUL_07_-_12MAY2015.pdf

[43] ——, "NDRRMC SitRep for TC Linfa," 2015. [Online]. Available: https://ndrrmc.gov.ph/attachments/article/2482/FINAL_REPORT_re_Severe_Tropical_Storm_EGAY_LINFA_as_of_02_-_07JUL2015.pdf

[44] ——, "NDRRMC SitRep for TC Ineng," 2015. [Online]. Available: https://ndrrmc.gov.ph/attachments/article/2536/FINAL_REPORT_re_Effects_of_Typhoon_INENG_GONI_18_-_23AUG2015.pdf

[45] ——, "NDRRMC SitRep for TC Mujigae," 2015. [Online]. Available: https://ndrrmc.gov.ph/attachments/article/2595/FINAL_REPORT_re_Effects_of_Tropical_Storm_KABAYAN_MUJIGAE_01_-_03OCT2015.pdf

[46] ——, "NDRRMC SitRep for TC Koppu," 2015. [Online]. Available: https://ndrrmc.gov.ph/attachments/article/2607/FINAL_REPORT_re_Preparedness_Measures_and_Effects_of_Typhoon_LANDO_KOPPU_as_of_14_-_21OCT2015.pdf

[47] ——, "NDRRMC SitRep for TC Melor," 2015. [Online]. Available: https://ndrrmc.gov.ph/attachments/article/2663/FINAL_REPORT_re_Effects_of_Typhoon_NONA_MELOR_as_of_12-17DEC2015.pdf

[48] ——, "NDRRMC SitRep for TC Onyok," 2015. [Online]. Available: https://ndrrmc.gov.ph/attachments/article/2665/FINAL_REPORT_re_Tropical_Depression_ONYOK_as_of_16-18DEC2015.pdf

[49] ——, "NDRRMC SitRep for TC Nida," 2016. [Online]. Available: https://ndrrmc.gov.ph/attachments/article/2858/Sitrep_No_05_re_Preparedness_Measures_and_Effects_of_Severe_Tropical_Storm_CARINA_as_of_01AUG2016_1700H_a.pdf

[50] ——, "NDRRMC SitRep for TC Meranti," 2016. [Online]. Available: https://ndrrmc.gov.ph/attachments/article/2913/SitRep_No_13_re_Preparedness_Measures_and_Effects_of_Typhoon_FERDIE_MERANTI_Covering_the_240600H_to_250600H_as_of_25SEP2016.pdf

[51] ——, "NDRRMC SitRep for TC Helen," 2016. [Online]. Available: https://ndrrmc.gov.ph/attachments/article/2922/SitRep_No_05_re_Preparedness_Measures_and_Effects_of_Typhoon_HELEN_MEGI_as_of_28SEP2016.pdf

[52] ——, "NDRRMC SitRep for TC Sarika," 2016. [Online]. Available: https://ndrrmc.gov.ph/attachments/article/2941/SitRep_No_08_re_Preparedness_Measures_and_Effects_of_Typhoon_KAREN_SARIKA_covering_the_period_190600H_to_200800H_as_of_20OCTOBER2016.pdf

[53] ——, "NDRRMC SitRep for TC Haima," 2016. [Online]. Available: https://ndrrmc.gov.ph/attachments/article/2946/Sitrep_No_09_re_Preparedness_Measures_and_Effects_of_Super_Typhoon_LAWIN_HAIMA_as_of_25OCT2016_0800H.pdf

[54] ——, "NDRRMC SitRep for TC Tokage," 2016. [Online]. Available: https://ndrrmc.gov.ph/attachments/article/2968/Sitrep_No_4_re_Preparedness_Measures_and_Effects_of_TS_Marce_TOKAGE_as_of_26NOV2016.pdf

[55] ——, "NDRRMC SitRep for TC Nock-Ten," 2016. [Online]. Available: https://ndrrmc.gov.ph/attachments/article/2975/Sitrep_No_10_re_Preparedness_Measures_and_Effects_of_TY_NINA_as_of_31DEC2016_0800H.pdf

[56] ——, "NDRRMC SitRep for TC Auring," 2017. [Online]. Available: https://ndrrmc.gov.ph/attachments/article/4154/1_NDRRMC_TD_AURING_2017.pdf

[57] ——, "NDRRMC SitRep for TC Crising_2," 2017. [Online]. Available: https://ndrrmc.gov.ph/attachments/article/4154/11_NDRRMC_TC_CRISING_2017.pdf

[58] ——, "NDRRMC SitRep for TC Pakhar," 2017. [Online]. Available: https://ndrrmc.gov.ph/attachments/article/4154/20_NDRRMC_TS_JOLINA_2017.pdf

[59] ——, "NDRRMC SitRep for TC Doksuri," 2017. [Online]. Available: https://ndrrmc.gov.ph/attachments/article/4154/22_NDRRMC_LANNIE_MARING_2017.pdf

[60] ——, "NDRRMC SitRep for TC Khanun," 2017. [Online]. Available: https://ndrrmc.gov.ph/attachments/article/4154/23_NDRRMC_STS_ODETTE_2017.pdf

[61] ——, "NDRRMC SitRep for TC Damrey," 2017. [Online]. Available: https://ndrrmc.gov.ph/attachments/article/4154/26_NDRRMC_RAMIL_2017.pdf

[62] ——, "NDRRMC SitRep for TC Haikui," 2017. [Online]. Available: https://ndrrmc.gov.ph/attachments/article/4154/27_NDRRMC_TS_SALOME_2017.pdf

[63] ——, “NDRRMC SitRep for TC Kirogi,” 2017. [Online]. Available: https://ndrrmc.gov.ph/attachments/article/4154/28_NDRRMC_TS_TINO_2017.pdf

[64] ——, “NDRRMC SitRep for TC Kai-Tak,” 2017. [Online]. Available: https://ndrrmc.gov.ph/attachments/article/4154/25_NDRRMC_TS_URDUJA_2017.pdf

[65] ——, “NDRRMC SitRep for TC Tembin,” 2017. [Online]. Available: https://ndrrmc.gov.ph/attachments/article/4154/29_NDRRMC_VINTA_2017.pdf

[66] ——, “NDRRMC SitRep for TC Bolaven,” 2018. [Online]. Available: https://ndrrmc.gov.ph/attachments/article/4153/1_NDRRMC_AGATON_2018.pdf

[67] ——, “NDRRMC SitRep for TC Sanba,” 2018. [Online]. Available: https://ndrrmc.gov.ph/attachments/article/4153/2_NDRRMC_BASYANG_2018.pdf

[68] ——, “NDRRMC SitRep for TC Mangkhut,” 2018. [Online]. Available: https://ndrrmc.gov.ph/attachments/article/4153/12_NDRRMC_OMPONG_2018.pdf

[69] ——, “NDRRMC SitRep for TC Yutu,” 2018. [Online]. Available: https://ndrrmc.gov.ph/attachments/article/4153/13_NDRRMC_Rosita_2018.pdf

[70] ——, “NDRRMC SitRep for TC Usagi,” 2018. [Online]. Available: https://ndrrmc.gov.ph/attachments/article/4153/14_NDRRMC_SAMUEL_2018.pdf

[71] ——, “NDRRMC SitRep for TC Usman,” 2018. [Online]. Available: https://ndrrmc.gov.ph/attachments/article/4153/15_NDRRMC_USMAN_2018.pdf

[72] ——, “NDRRMC SitRep for TC Amang,” 2019. [Online]. Available: https://ndrrmc.gov.ph/attachments/article/4183/5_NDRRMC_AMANG_2019.pdf

[73] ——, “NDRRMC SitRep for TC Chedeng,” 2019. [Online]. Available: https://ndrrmc.gov.ph/attachments/article/4183/8_NDRRMC_CHEDENG_2019.pdf

[74] ——, “NDRRMC SitRep for TC Danas,” 2019. [Online]. Available: https://ndrrmc.gov.ph/attachments/article/4183/17_NDRRMC_FALCON_2019.pdf

[75] ——, “NDRRMC SitRep for TC Marilyn,” 2019. [Online]. Available: https://ndrrmc.gov.ph/attachments/article/3895/SitRep_No_09_re_Preparedness_Measures_and_Effects_of_SW_Monsoon_Enhanced_by_TD_MARILYN_02OCT2019_6AM.pdf

[76] ——, “NDRRMC SitRep for TC Kalmaegi,” 2019. [Online]. Available: https://ndrrmc.gov.ph/attachments/article/4183/28_NDRRMC_RAMON_2019.pdf

[77] ——, “NDRRMC SitRep for TC Kammuri,” 2019. [Online]. Available: https://ndrrmc.gov.ph/attachments/article/4183/32_NDRRMC_TISOY_2019.pdf

[78] ——, “NDRRMC SitRep for TC Phanfone,” 2019. [Online]. Available: https://ndrrmc.gov.ph/attachments/article/4183/35_NDRRMC_URSULA_2019.pdf

[79] ——, “NDRRMC SitRep for TC Vongfong,” 2020. [Online]. Available: https://ndrrmc.gov.ph/attachments/article/4048/Update_Sitrep_no_15_re_Preparedness_Measures_and_Effects_of_TY_AMBO_Issued_on_27May2020_12NN.pdf

[80] ——, “NDRRMC SitRep for TC Ofel,” 2020. [Online]. Available: https://ndrrmc.gov.ph/attachments/article/4128/Update_Sitrep-5-TD-OFEL.pdf

[81] ——, “NDRRMC SitRep for TC Saudel,” 2020. [Online]. Available: https://ndrrmc.gov.ph/attachments/article/4129/SitRep_No_07_Preparedness_Measures_and_Effects_of_TY_Pepito_as_of_26Oct2020_8AM.pdf

[82] ——, “NDRRMC SitRep for TC Molave,” 2020. [Online]. Available: https://ndrrmc.gov.ph/attachments/article/4130/SitRep_no_11_re_Typh00n_Quinta_as_of_09NOV2020.pdf

[83] ——, “NDRRMC SitRep for TC Goni,” 2020. [Online]. Available: https://ndrrmc.gov.ph/attachments/article/4135/SitRep_no_12_re_STY_ROLLY_as_of_11NOV2020.pdf

[84] ——, “NDRRMC SitRep for TC Vamco,” 2020. [Online]. Available: https://ndrrmc.gov.ph/attachments/article/4138/SitRep_no_28_re_TY_ULYSSES_as_of_10DEC2020.pdf

[85] ——, “NDRRMC SitRep for TC Krovanh,” 2020. [Online]. Available: https://ndrrmc.gov.ph/attachments/article/4139/SitRep_no_07_re_TD_VICKY_as_of_26DEC2020.pdf
